# A Structural Model for Binding of the Serine-Rich Repeat Adhesin GspB to Host Carbohydrate Receptors

**DOI:** 10.1371/journal.ppat.1002112

**Published:** 2011-07-07

**Authors:** Tasia M. Pyburn, Barbara A. Bensing, Yan Q. Xiong, Bruce J. Melancon, Thomas M. Tomasiak, Nicholas J. Ward, Victoria Yankovskaya, Kevin M. Oliver, Gary Cecchini, Gary A. Sulikowski, Matthew J. Tyska, Paul M. Sullam, T. M. Iverson

**Affiliations:** 1 Department of Pharmacology, Vanderbilt University Medical Center, Nashville, Tennessee, United States of America; 2 Vanderbilt Institute of Chemical Biology, Nashville, Tennessee, United States of America; 3 Department of Medicine, Veterans Affairs Medical Center and the University of California, San Francisco, California, United States of America; 4 Department of Medicine, Harbor-UCLA Medical Center, Torrance, California, United States of America; 5 Department of Chemistry, Vanderbilt University, Nashville, Tennessee, United States of America; 6 Molecular Biology Division, Veterans Affairs Medical Center, San Francisco, California, United States of America; 7 Department of Biochemistry & Biophysics University of California, San Francisco, California, United States of America; 8 Department of Cell and Developmental Biology, Vanderbilt University Medical Center, Nashville, Tennessee, United States of America; 9 Department of Biochemistry, Vanderbilt University Medical Center, Nashville, Tennessee, United States of America; University of California San Diego, United States of America

## Abstract

GspB is a serine-rich repeat (SRR) adhesin of *Streptococcus gordonii* that mediates binding of this organism to human platelets via its interaction with sialyl-T antigen on the receptor GPIbα. This interaction appears to be a major virulence determinant in the pathogenesis of infective endocarditis. To address the mechanism by which GspB recognizes its carbohydrate ligand, we determined the high-resolution x-ray crystal structure of the GspB binding region (GspB_BR_), both alone and in complex with a disaccharide precursor to sialyl-T antigen. Analysis of the GspB_BR_ structure revealed that it is comprised of three independently folded subdomains or modules: 1) an Ig-fold resembling a CnaA domain from prokaryotic pathogens; 2) a second Ig-fold resembling the binding region of mammalian Siglecs; 3) a subdomain of unique fold. The disaccharide was found to bind in a pocket within the Siglec subdomain, but at a site distinct from that observed in mammalian Siglecs. Confirming the biological relevance of this binding pocket, we produced three isogenic variants of *S. gordonii*, each containing a single point mutation of a residue lining this binding pocket. These variants have reduced binding to carbohydrates of GPIbα. Further examination of purified GspB_BR_-R484E showed reduced binding to sialyl-T antigen while *S. gordonii* harboring this mutation did not efficiently bind platelets and showed a significant reduction in virulence, as measured by an animal model of endocarditis. Analysis of other SRR proteins revealed that the predicted binding regions of these adhesins also had a modular organization, with those known to bind carbohydrate receptors having modules homologous to the Siglec and Unique subdomains of GspB_BR_. This suggests that the binding specificity of the SRR family of adhesins is determined by the type and organization of discrete modules within the binding domains, which may affect the tropism of organisms for different tissues.

## Author Summary

The binding of bacteria to human platelets is thought to be important for development of infective endocarditis, a life-threatening infection of the cardiovascular system. *Streptococcus gordonii* is a leading cause of endocarditis. This pathogen uses a protein called GspB to attach to carbohydrates on human platelets. While this binding interaction appears to be mediated by a specific, contiguous domain within GspB, little is known about the molecular details of the interaction between GspB and the carbohydrate receptors on its human host. We therefore determined the crystal structure of the region of GspB that binds to platelet carbohydrates, both alone and in complex with a synthetic carbohydrate receptor. Using this structure as a guide, we were able to produce three strains of *S. gordonii* that lacked the ability to bind to platelet carbohydrates. One of these isogenic variants was studied more in-depth and lacked the ability to bind to human platelets *in vitro* and was reduced in virulence when tested *in vivo*. These studies provide the first structural information detailing the molecular interactions between any serine-rich repeat adhesin and its host receptor, and identify how different, related adhesins may have evolved different specificities for host receptors.

## Introduction

**Figure 1:**
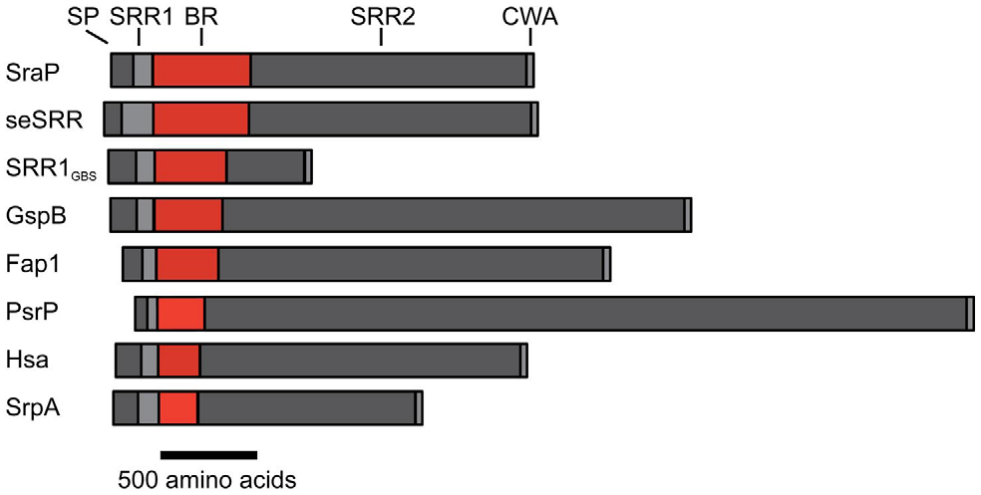
Schematic of the SRR adhesins. Selected SRR adhesins are aligned based on the N-terminus of their binding regions, with the largest binding region at the top and the smallest binding region at the bottom. The length of each domain and the entire protein is drawn to-scale. SP = signal peptide (*dark gray*), SRR1 = first serine rich repeat (*light gray*), BR = binding region (*red*), SRR2 = second serine rich repeat (*dark gray*), CWA = cell wall anchoring motif (*light gray*). The SRR adhesins used in this figure are *S. aureus* SraP (SraP); *S. epidermidis* SRR1 (seSRR), *S. agalactiae* SRR1 (SRR1_GBS_), *S. gordonii* strain M99 GspB (GspB), *S. parasanguinis* Fap1 (Fap1), *S. pneumoniae* PsrP (PsrP), *S. gordonii* strain Challis Hsa (Hsa), and *S. sanguinis* SrpA (SrpA).

The serine-rich repeat (SRR) glycoproteins of Gram-positive bacteria are an expanding family of microbial adhesins and virulence factors [1]–[6]. These proteins consist of a distinctive signal sequence and export-targeting region at the N-terminus, a short SRR region (∼50–170 amino acids), a ligand binding region, a second lengthy SRR region (∼400–4000 amino acids), and a cell wall anchoring motif at the C-terminus (**Fig. 1**) [1], [2], [4], [6]–[9]. The binding regions of the SRR glycoproteins contain significant sequence variation, which appears to account for their broad range of binding targets, including platelet membrane and salivary glycoproteins [1], [4], [9]–[11], endothelial cells [12], epithelial cells [13], erythrocytes [14], [15], and keratins [16], [17]. Expression of SRR proteins has been associated with increased virulence in several animal models of infection, including endocarditis [6], [18], meningitis [12], pneumonia [5], and bacteremia [7], [19].

A number of bacterial surface components have been shown to mediate platelet binding *in vitro*, either by interacting directly with receptors on the platelet membrane, or via bridging molecules, such as fibrinogen [20]–[29]. The contribution of these interactions to virulence, however, has been assessed for relatively few of these adhesins. Previous studies have focused on the molecular basis for the SRR adhesin mediated binding of gram-positive bacteria to human platelets, and the role of this process in the pathogenesis of infective endocarditis. This interaction appears to be important for the attachment of blood-borne bacteria to platelets on the surface of damaged cardiac valves, thereby initiating infection. In addition, the subsequent deposition of platelets onto the infected endocardium may be due in part to bacterium-platelet binding, resulting in the formation macroscopic vegetations, which are the hallmark lesions of this disease [30]. Three of the SRR proteins (GspB of *Streptococcus gordonii* strain M99, Hsa of *S. gordonii* strain Challis, and SrpA of *Streptococcus sanguinis* strain SK36) bind human platelets through their interaction with glycocalicin, which is the carbohydrate-rich extracellular portion of the platelet membrane glycoprotein GPIbα [9], [11]. While the specific carbohydrate receptor for SrpA has not yet been identified, GspB and Hsa recognize sialylated trisaccharides [1], [11], [31], [32]. Dot blot assays using immobilized carbohydrates have demonstrated that GspB has high fidelity for sialyl-T antigen (i.e. NeuAcα(2–3)Galβ(1–3)GalNAc), one of the major carbohydrates on GPIbα [11], while Hsa binds to glycocalicin via either sialyl-T antigen or sialyllactose (Neu5Acα(2–6)Galβ(1–4)Glc) [31], [32]. Binding of these SRR adhesins to platelets is a high affinity process, with the interaction between GspB and glycocalicin having a K_D_ of 2.38×10^−8^ M [11] and appears to be a major factor in the pathogenesis of infective endocarditis, since the loss of GspB or Hsa expression results in a marked reduction in virulence [33].

Structural information can enhance the understanding of the determinants of binding specificity. Here, we report the high-resolution crystal structure of GspB_BR_, both alone and in complex with the disaccharide α-2,3-sialyl (1-thioethyl)galactose, a precursor to synthetically-produced sialyl-T antigen. From these structures, we identified that a subdomain of GspB_BR_ resembling mammalian sialic acid binding proteins binds to α-2,3-sialyl (1-thioethyl)galactose. Site directed mutagenesis and *in vivo* studies in a rat model of infective endocarditis verified that this carbohydrate binding site within GspB_BR_ mediates binding of *S. gordonii* strain M99 to sialyl-T antigen, the host carbohydrates of the platelet membrane receptor GPIbα, and intact platelets, and that this interaction is important for virulence. Our analysis of the structure further identified that GspB_BR_ contains an unusually modular fold, prompting us to re-analyze the sequences of the SRR family of adhesins. We determined that other structurally uncharacterized SRR adhesins also contain modules within their binding regions, suggesting that particular subdomains may be included, removed, or interchanged, manifesting in the broad range of binding partners observed in the family.

## Results

### Overall structure of GspB_BR_

**Figure 2:**
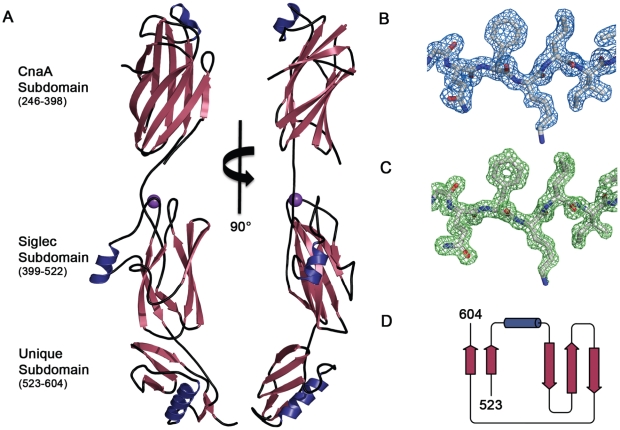
Structure of GspB_BR_. A. Two views of the structure of GspB_BR_ separated by a 90° rotation. The N-terminus of the binding region is located at the top of the figure and the C-terminus is located at the bottom. β-Strands are colored *magenta*, α-helices are colored *blue*, and loops are colored *black*. A cation bound within the Siglec domain is shown as a *purple sphere*. B. Representative experimental electron density (*blue mesh*) at 2.0 Å resolution, contoured at 1.0 σ, and depicted superpositioned onto residues 515–520 of the final model. C. A m|F_o_|−d|F_c_| omit electron density map at 1.4 Å resolution (*green mesh*) calculated in REFMAC5 [65] after the removal of the model contoured at 3.0 σ and depicted superpositioned onto residues 515–520 of the final model. D. Folding diagram of the Unique subdomain. β-Strands are colored *magenta* and the α-helix is colored *blue*.

**Figure 3:**
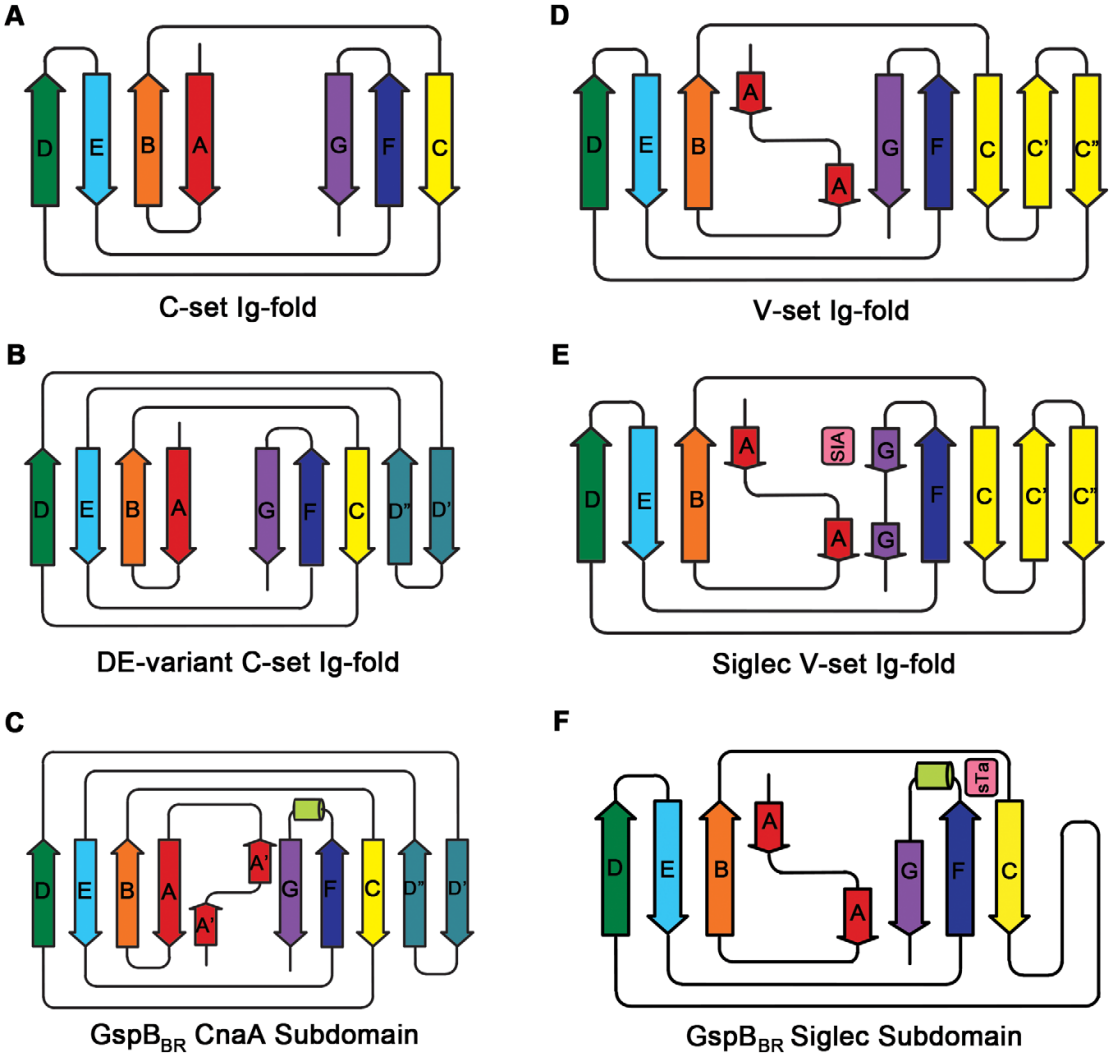
Ig-fold topologies. A. Topology diagram of a C-set Ig-fold. B. Topology diagram of the DE-variant of a C-set Ig-fold identified in MSCRAMMs [36]. C. Topology diagram of the GspB_BR_ CnaA subdomain. D. Topology diagram of a V-set Ig-fold. E. Topology diagram of a Siglec [45] showing the binding location of the sialylated carbohydrate in *pink*. F. Topology diagram of the GspB_BR_ Siglec subdomain showing the binding location of the sialyl-T antigen in pink.

We determined the structure of GspB_BR_ to 1.4 Å resolution using the method of Multiwavelength Anomalous Dispersion from a single Dy^3+^ derivative (**Fig. 2** **,** **Table 1** **,** **Table 2**). GspB_BR_ folds into an elongated rod, with dimensions of ∼130 Å×30 Å×30 Å. This rod is comprised of three apparently independently-folded subdomains arranged in a linear fashion, like beads on a string. The secondary structure of each of the three subdomains is predominated by β-strands. Interestingly, the first two subdomains are organized around core folds that resemble those found within the eukaryotic immunoglobulin (Ig) superfamily (**Fig. 3**). Ig-folds have previously been identified in prokaryotic proteins [34]–[41], and it has been noted that some of these bacterial proteins with Ig-folding topologies contain sequence similarity to their eukaryotic counterparts, while the others lack the residues conserved in the core of eukaryotic proteins with Ig-folds. GspB_BR_ has homology with bacterial proteins that do not contain detectable sequence similarity to eukaryotic proteins with Ig-folds, lacking even the cysteines that normally form a disulfide bond.

**Table 1:** X-ray data collection and refinement statistics.

PDB Accession Number	3QC5	3QC6	3QD1
	Native	Native2	Disaccharide
Space group	P2_1_2_1_2_1_	P2_1_2_1_2_1_	P2_1_
Wavelength (Å)	0.97856	1.03034	1.07806
Beamline	LS-CAT ID-21-F	SSRL 11-1	LS-CAT ID-21-D
Resolution (Å)	50-1.40	50-1.90	50-1.90
Unit-cell a,b,c (Å)	33.7, 96.8, 100.2	33.3, 86.7, 117.9	69.9, 34.0, 83.4
No. of measured reflections	223296	84235	97430
No. of unique reflections	60816	26193	29986
Multiplicity	3.7(2.0)^1^	3.2(2.9)	3.2(2.6)
I_mean_/σ(I)	19.8(2.9)	16.8(3.9)	17.4(4.8)
Completeness (%)	92.7(54.1)	93.8(92.4)	96.3(93.0)
R_sym_ 2 (%)	4.1(24.4)	6.8(35.3)	8.9(35.6)
R_work_ 3/R_free_ 4 (%)	14.2/17.2	21.5/26.1	17.8/21.4
No. of reflections in test set	2543	1265	1477
No. of protein atoms	352	351	359
No. of water atoms	400	231	212
RMS deviation bonds (Å)	0.012	0.013	0.12
RMS deviation angles (°)	1.38	1.36	1.30

1 Numbers in parentheses indicate values for the highest resolution bin.

2 R_sym_ = ∑[I_i_−<I>](100)/∑[<I>] where i is ith measurement and <I> is the weighted mean of I.

3 R_work_ = ∑||F_obs_|−|F_calc_||/∑|F_obs_|.

4 R_free_ is calculated using the same equation as for R_work_ but using only the reflections randomly omitted from refinement reserved in the test set of the data.

**Table 2:** Derivative data collection and phasing statistics.

	Peak	Inflection	Remote
Space group	P2_1_2_1_2_1_	P2_1_2_1_2_1_	P2_1_2_1_2_1_
Wavelength (Å)	1.59083	1.59122	1.02463
Beamline	SSRL 9-2	SSRL 9-2	SSRL 9-2
Resolution (Å)	50-1.98	50-1.98	50-1.55
Unit-cell a,b,c (Å)	35.5, 98.5, 99.1	34.5, 98.5, 99.1	34.5, 98.5, 99.0
No. of measured reflections	203951	206064	431338
No. of unique reflections	23918	23929	48691
Multiplicity	8.5(8.1)^1^	8.6(8.1)	8.9(8.3)
I_mean_/σ(I)	31.2(19.3)	31.2(18.4)	27.7(6.2)
Completeness (%)	98.0(96.2)	98.0(95.6)	97.2(99.6)
R_sym_ 2 (%)	8.7(17.9)	8.0(17.7)	6.1(35.9)
Phasing Power^3^	4.1	3.6	2.1

1 Numbers in parentheses indicate values for the highest resolution bin.

2 R_sym_ = ∑[I_i_−<I>](100)/∑[<I>] where i is ith measurement and <I> is the weighted mean of I.

3 Phasing power is defined as the mean value of the heavy atom structure factor divided by the lack of closure error.

The overall Figure of Merit was 0.83.

Several topology variants of Ig-folds have been characterized. A structural homology search using the EMBL DaliLite server [42] identified that the N-terminal subdomain of GspB_BR_ contains a folding topology, strand inserts, and inter-sheet angle reminiscent of the DE-variant of the Ig-fold [36] (**Fig. 3A–C**). This Ig-fold topology is found in prokaryotic proteins and was first identified within the A-region of the *Staphylococcus aureus* CNA protein [35]. CNA belongs to the family of microbial surface components recognizing adhesive matrix molecules (MSCRAMMs) of Gram-positive pathogens [34]–[36]. Given its structural similarity, this N-terminal subdomain of GspB_BR_ will be termed the CnaA subdomain. The highest structural similarity between the CnaA subdomain of GspB_BR_ and any other structurally characterized protein is to the C-terminal subdomain of the binding region of SRR adhesin Fap1 from *Streptococcus parasanguinis* (Fap1_NR_-β) [43]. The RMS deviation of the structural alignment between the CnaA subdomain of GspB_BR_ and the Fap1_NR_-β subdomain is 2.5 Å for 200 C_α_ atoms.

Surprisingly, a structural homology search using EMBL DaliLite [42] identified that the second subdomain contained a topology and strand inserts reminiscent of the V-set Ig fold adopted by eukaryotic sialic acid binding immunoglobulin-like lectins [44]–[46] (Siglecs; **Fig. 3D–F**). However, the C′ and C″ strands normally inserted into the V-set Ig-fold are instead replaced by a long loop inserted at the same location (**Fig. 3D–F**). The RMS deviation of the structural alignment between the second subdomain of GspB_BR_ and Siglec-5 is 2.9 Å for 210 C_α_ atoms despite only 6% sequence identity. Like GspB, Siglecs bind carbohydrate receptors. Accordingly, the second domain is termed the Siglec subdomain. This subdomain of GspB_BR_ contained electron density consistent with a cation-binding site (see **Supporting Text S1, Fig. S1, Supporting Protocol S1**). This was rather unexpected since Siglecs themselves do not bind cations in the carbohydrate-binding domain. The seven-coordinate number suggests that under physiological conditions, this should be a Ca^2+^ binding site. We explored the role of Ca^2+^ and other cations for the binding of *S. gordonii* strain M99 to glycocalicin. However, metal depletion, metal substitution, and site directed mutagenesis of the residues coordinating the ion were all consistent with the cation not being essential for carbohydrate receptor recognition (data not shown).

While still predominated by β-strands, the C-terminal subdomain of GspB_BR_ does not contain an Ig-fold. In fact, a structural homology search using DaliLite [42] did not identify any proteins of known structure with significant similarity to this C-terminal subdomain (**Fig. 2D**). As a result, it will be termed the Unique subdomain.

### Identification of the receptor binding site

**Figure 4:**
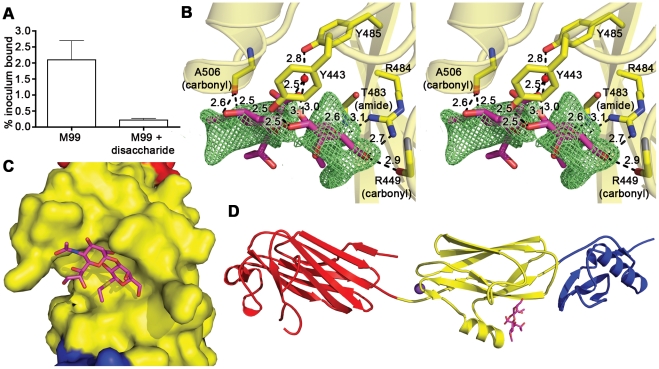
Identification of the receptor binding site. **A.** Inhibition of *S. gordonii* strain M99 binding to immobilized glycocalicin by the α-2,3-sialyl (1-thioethyl)galactose disaccharide. Binding was assessed in the absence or presence of 44 mM disaccharide, and is expressed as the percent of input bacteria that remained bound to glycocalicin after repeated washing of the microtiter wells (mean ± standard deviation). B. Stereo view of the disaccharide binding site superpositioned with m|F_o_|−d|F_c_| omit electron density (*green mesh*) calculated in REFMAC5 [65] and contoured to 2.5 σ after the removal of disaccharide from the model. C. A space filling model of GspB_BR_ with bound disaccharide (*stick model*) demonstrates that the carbohydrate receptor binds within a pocket on the surface of the protein. The protein surface is colored by subdomain with the Siglec subdomain in *yellow* and the Unique subdomain in *blue*. The stick model of the disaccharide has carbon atoms colored *magenta*, oxygen atoms colored *red*, the nitrogen atom colored *blue*, and the sulfur atom colored *yellow*. The model is rotated 50° along the y-axis and 40° along the x-axis as compared to panel B. D. Overview of the GspB_BR_ structure highlighting the location of the disaccharide binding site in the Siglec subdomain. The model is rotated 90° along the x-axis as compared to panel B. The disaccharide is shown with *magenta* bonds and the cation is highlighted as a *purple sphere*.

We sought to identify the details of the interaction between GspB_BR_ and the host receptor sialyl-T antigen. Free sialyl-T antigen is a rare reagent that is not commercially available and is challenging to synthesize. Therefore, we developed a 4-step synthesis for α-2,3-sialyl (1-thioethyl)galactose (NeuAcα(2–3)(1-CH_3_CH_2_S)Galβ) (see **Supporting Protocol S1; Fig. S2**) which is the disaccharide truncation of and a synthetic precursor to sialyl-T antigen (NeuAcα(2–3)Galβ(1–3)GalNAc). To confirm that α-2,3-sialyl (1-thioethyl)galactose binds to GspB_BR_, we assessed the ability of this disaccharide to inhibit the binding of *S. gordonii* to glycocalicin. In the presence of 44 mM α-2,3-sialyl (1-thioethyl)galactose, binding of *S. gordonii* to glycocalicin was reduced by 90% (**Fig. 4A**). Given the structural similarity to the native host receptor, this strongly suggests that α-2,3-sialyl (1-thioethyl)galactose competes directly for the sialyl-T antigen binding site.

**Figure 5:**
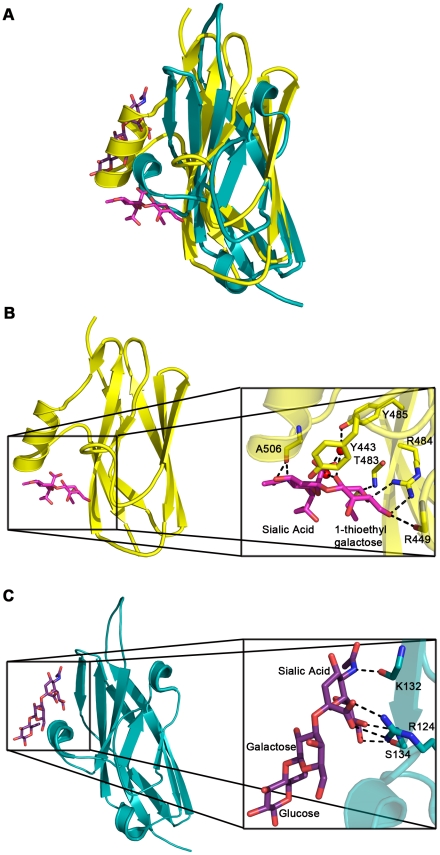
Comparison of carbohydrate binding in the Siglec subdomain of GspB_BR_ and Siglec-5. A. Overlay of the Siglec subdomain of GspB_BR_ and Siglec-5. Siglec-5 is colored *teal* with the carbons of 3′ sialyllactose colored *purple*. The Siglec subdomain of GspB_BR_ is colored *yellow* with the carbons of α-2,3-sialyl (1-thioethyl)galactose colored in *magenta*. In this panel, the Siglec carbohydrate binding site is occupied by a helix in the GspB_BR_ Siglec subdomain therefore eliminating the possibility that this could be a second binding site for GspB_BR_. B & C. Comparison of the location and details of the carbohydrate binding site in (B) GspB_BR_ with that of (C) Siglec-5 (PDB entry 2ZG3; [45]). The Siglec subdomain of GspB_BR_ is colored *yellow* with the carbons of α-2,3-sialyl (1-thioethyl)galactose colored in *magenta* and Siglec-5 is colored *teal* with the carbons of 3′ sialyllactose colored *purple*. While Siglec-5 is used as an example, it is noted that for all structurally characterized Siglecs (Siglec-1 (aka sialoadhesin); PDB entry 1QFO; [44]), Siglec-5 (2ZG3; [45]), and Siglec-7 (PDB entry 2HRL; [74])), the sialic acid binding pocket is found at the same location.

We determined the co-crystal structure of α-2,3-sialyl (1-thioethyl)galactose in complex with GspB_BR_. New electron density consistent with bound disaccharide (**Fig. 4B**) was apparent within a defined pocket (**Fig. 4C**) located at the N-terminus of the C strand and C-terminus of the F strand in the Siglec domain (**Fig. 3F**) of the Siglec subdomain (**Fig. 4D**). While the similarity of the fold of the Siglec subdomain to mammalian Siglecs might predict a similar binding pocket, both the overall location of the α-2,3-sialyl (1-thioethyl)galactose binding site on the domain and the specific contacts between GspB_BR_ to the carbohydrate differ from that of structurally characterized mammalian Siglecs [44]–[46] (**Fig. 5**). In fact, in GspB_BR_ a helix is positioned in the location where carbohydrates bind to Siglecs, precluding the use of a structurally similar binding site. Instead, the local secondary structure surrounding the α-2,3-sialyl (1-thioethyl)galactose binding site resembles a β-grasp domain, which is a motif that commonly binds the α-2,3-linkage of sialic acid based multivalent carbohydrates [47]–[49]. In GspB_BR_, the backbone carbonyls of Ala506 and Arg449, the Nη1 and Nη2 of Arg484, the backbone amide nitrogen of Thr483 and the side chain hydroxyl of Tyr443 make direct contacts to the α-2,3-sialyl (1-thioethyl)galactose disaccharide, while the side chain hydroxyl of Tyr485 makes water-mediated contacts (**Fig. 4B** **,** **Table 3**).

**Table 3:** Contacts at the receptor binding site.

Ligand Atom	Protein Atom	Distance (Å)
OAW	Ala506 O	2.6
NAO	Ala506 O	2.5
OAT	HOH213*	2.5
OAP	HOH213*	3.1
O2	HOH213*	2.5
O2	HOH199*	2.9
O3	Tyr443 OH	2.5
SAC	Thr483 N	2.6
O5	Arg484 Nη1	3.1
O6	Arg484 Nη2	2.7
O6	Arg449 O	2.9

*Water molecules make water mediated contacts to the protein and are not just solvating.

A comparison of the structure of GspB_BR_ determined with and without the disaccharide did not show significant structural changes localized within the binding pocket, which suggests that it is pre-formed. Thus, while the physiologically-relevant receptor trisaccharide sialyl-T antigen is a rare reagent, we can qualitatively suggest its binding location by modeling the third carbohydrate onto α-2,3-sialyl (1-thioethyl)galactose. In our model, all three carbohydrates of the host receptor fit into a pre-formed binding pocket on the Siglec subdomain, with the third carbohydrate of sialyl-T antigen extending toward the Unique subdomain (**Fig. S3**).

**Figure 6:**
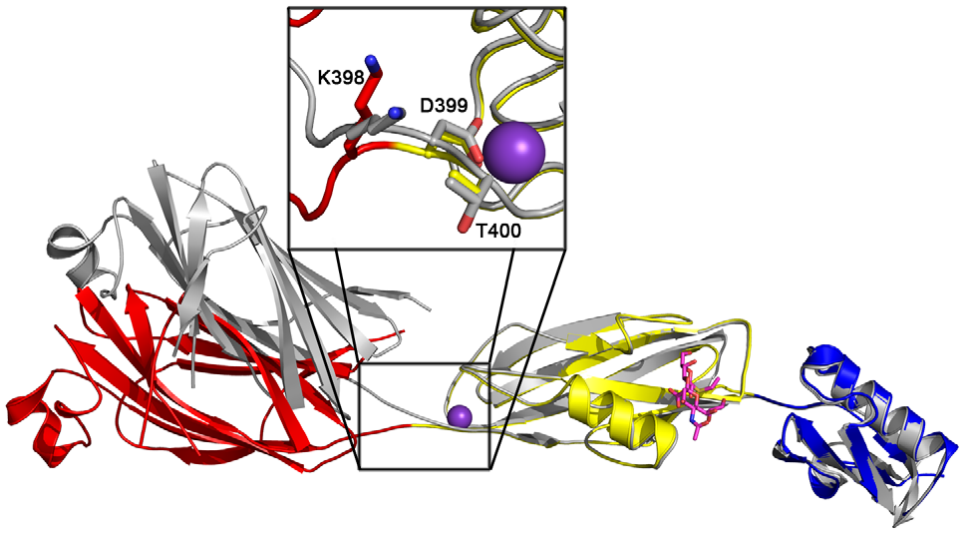
Interdomain Angle. Differences in interdomain angles between structures of GspB_BR_. The structures of the Siglec and Unique subdomains of unbound and disaccharide-bound GspB_BR_ were aligned using Dyndom [69]. The unbound structure is colored in *silver*, and the structure in complex with α-2,3-sialyl (1-thioethyl) galactose is colored by subdomain with the CnaA subdomain in *red*, the Siglec subdomain in *yellow* and the Unique subdomain colored in *blue*, and cation colored *purple*. The side chains of hinge residues Lys398, Asp399, and Thr400 are highlighted in the boxed inset. The difference in interdomain angle between the two structures is a 40°. A movie morphing these states is seen in **Video S1**.

While disaccharide binding to GspB_BR_ did not appear to induce obvious changes in conformations of side chains within the binding pocket, the interdomain angle between the CnaA and Siglec subdomains unexpectedly straightened by 40° (**Fig. 6**; **Video S1**). The change in orientation between the CnaA and Siglec subdomains involves rotation around a single hinge consisting of residues Lys398-Asp399-Thr400, where the side chain Asp399 is one of the ion coordinating residues.

### Functional relevance of the carbohydrate binding site

**Figure 7:**
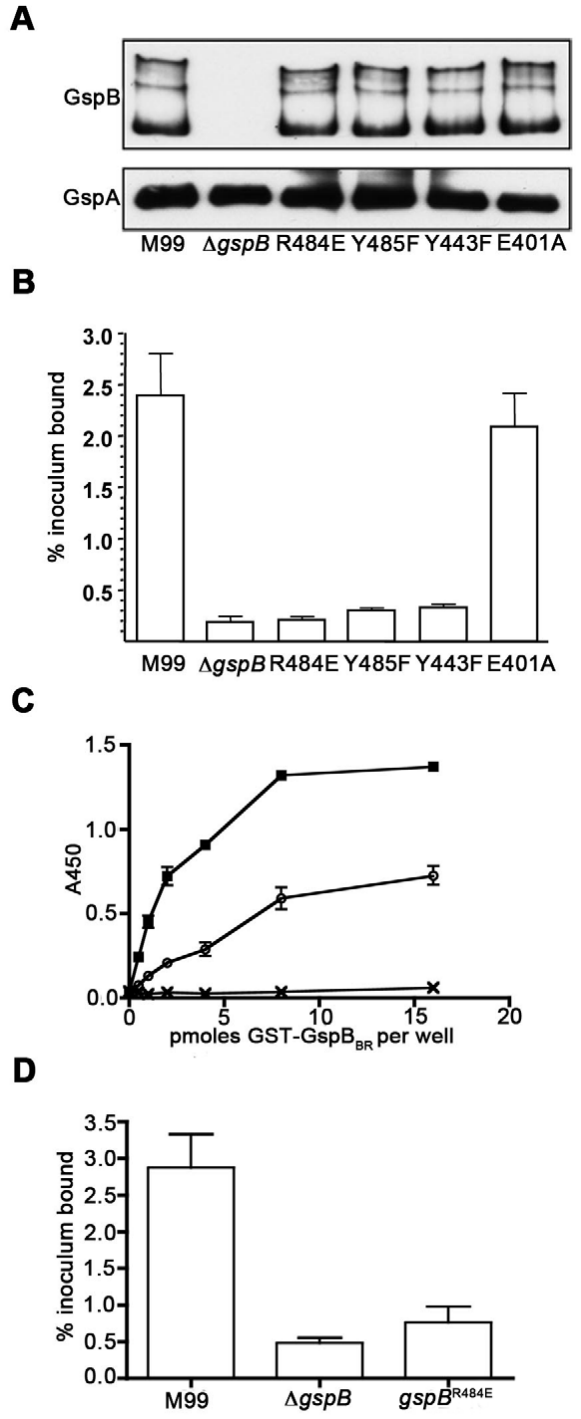
Amino acid substitutions in the carbohydrate binding site of GspB_BR_. A. Expression of GspB and variant proteins on the *S. gordonii* cell surface. Peptidoglycan-linked proteins were extracted from the bacterial cell surface and analyzed by western blotting using a polyclonal anti-GspB serum (upper panel). The nitrocellulose membrane was then re-probed using a polyclonal anti-GspA serum (lower panel) in order to assess the relative efficiency of protein extraction and protein loading (GspA is another LPXTG-linked protein that is expressed by *S. gordonii* M99). B. Binding of *S. gordonii* strain M99 and derivative strains to glycocalicin immobilized in microtiter wells. Binding is expressed as the percent of input bacteria that remained bound to glycocalicin after repeated washing of the wells (mean ± standard deviation). The labels of each column indicate the sequence of the protein expressed in *S. gordonii*, with M99 indicating wild type, Δ*gspB* indicating a strain where the gene encoding GspB has been deleted, and remaining columns indicating the amino acid substitution present in the protein. E401 is a control residue not located near the binding pocket. C. Binding of sialyl-T antigen to purified GST-GspB_BR_. The indicated amounts of GST or GST-GspB_BR_ wild-type (wt) and variant (R484E) proteins were immobilized in microtiter wells, and the binding of biotinylated sialyl-T antigen to each protein was detected by using peroxidase-conjugated streptavidin along with a chromogenic peroxidase substrate. Binding is expressed as the mean ± standard deviation (n = 3). The o represents purified GST-GspB_BR_-R484E, the x represents purified GST, and the 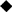 represents purified GST-GspB_BR_. D. Binding of M99 and derivative strains to human platelets. Binding is expressed as the percent of input bacteria that remained bound to platelets after repeated washing of the wells (mean ± standard deviation).

To confirm that this crystallographically-identified α-2,3-sialyl (1-thioethyl)galactose binding site is indeed important for GspB-mediated binding to GPIbα, we generated four isogenic variants of strain M99 with point mutations within the Siglec subdomain of GspB (**Table 4**). Three of these variants contained mutations within the crystallographically-identified binding site (Y443F, R484E and Y485F), while a control mutation (E401A) was located within the Siglec domain, but away from the receptor binding site. Importantly, none of the point mutations affected surface expression of GspB (**Fig. 7A**). Each of these isogenic variants had an 86% or greater decrease in binding to GPIbα *in vitro*, as compared with the parent strain (*p*<0.001) (**Fig. 7B**). This decrease is comparable to that of the *gspB* null mutant. In contrast, binding by the E401A variant was not significantly different from that of M99 (*p* = 0.44).

**Table 4:** Strain names with descriptions.

Strain name	Description
M99	*S. gordonii* strain M99 wild-type
PS2116	*S. gordonii* strain M99 *gspB* ^R484E^
PS846	*S. gordonii* strain M99Δ*gspB*
PS2161	5′*gspB::spec* control strain

**Figure 8:**
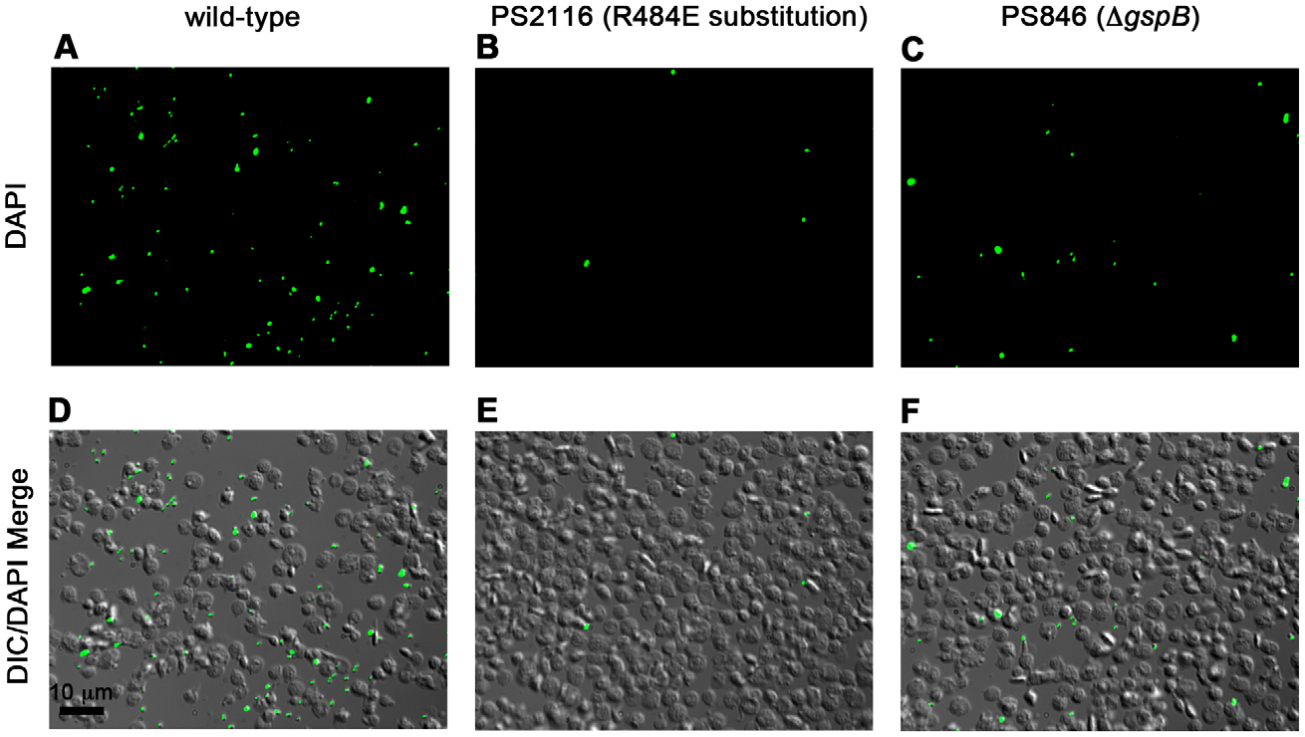
Binding of wild type *S. gordonii* strain M99, PS2116 (strain M99 with *gspb* harboring the R484E substitution), and PS846 (Δ*gspB*) to platelets. Fixed platelets were adhered to poly-L-lysine coated cover slips, quenched, and bound to live bacteria stained with DAPI. A–C. Fluorescent images of each strain with DAPI stain. D–F. Merge between the DIC image and the DAPI image to show relative number of platelets.

We then selected the R484E mutation for more detailed study. The purified GST-GspB_BR_ fusion protein harboring the R484E substitution exhibited a marked decrease in binding to biotinylated sialyl-T antigen (**Fig. 7C**). The R484E substitution also resulted in reduced binding to human platelets *in vitro* as assessed by quantifying the amount of input inoculum bound to fixed platelets (**Fig. 7D**). We next visualized platelets by Differential Interference Contrast (DIC) and fluorescence microscopy and quantified the number of platelets with surface-bound bacteria. In the presence of the DAPI-labeled PS846 (M99Δ*gspB*) or the PS2116 (M99 *gspb* ^R484E^) strain, substantially fewer platelets had bacteria bound as compared to platelets in the presence of wild-type *S. gordonii* strain M99 (**Fig. 8** **,** **Table 5**). It should be noted that while the platelet numbers were normalized when placed onto the cover slip, during the experiment the wild type bacteria seemed to form microscopic aggregates with the platelets (data not shown) but this was not observed when platelets were mixed with strains PS2116 or PS846. This confirms the importance of this residue for carbohydrate binding.

**Table 5:** Binding of three variants of *S. gordonii* to human platelets.

Strain	% of Platelets with Bacteria Bound (Mean ± S.D.)
M99	32.0±8.7
PS2116	3.0±1.7*
PS846	7.0±1.7*

*p = 0.01 as compared with M99.

Note: The difference in binding between the PS2116 and PS846 strains were not statistically significant.

### Impact of Siglec-mediated binding on virulence

It has previously been demonstrated that loss of GspB expression results in a marked decrease in virulence, as measured by a rat model of infective endocarditis [18]. To assess whether binding via the Siglec subdomain to host carbohydrate receptors contributes to virulence, we examined the impact of the R484E substitution on the ability of *S. gordonii* strain M99 to produce endocardial infection. We first compared the relative virulence of M99 with strain PS2116, (M99 *gspb* ^R484E^). Catheterized rats were simultaneously infected intravenously with 2×10^−5^ CFU of M99 and PS2116. After 72 h, the animals were sacrificed and the relative levels of bacteria within tissues determined. Animals co-infected with M99 and PS2161 (which carries the *spec* resistance marker just upstream of *gspB*) served as controls. When assessed at the above time-point, animals co-infected with M99 and PS2116 had significantly reduced densities of the mutant strain within vegetations (mean ± S.D. = 6.81±1.70 log_10_ CU/g. veg.) as compared with parental strain M99 (7.47±1.69 log_10_ CFU/g. veg; *P*<0.02). Loss of sialyl-T antigen binding was also associated with significantly reduced bacterial densities within kidneys and spleens (*P*<0.02 and *P*<0.001, respectively; **Table 6**). In parallel studies, no differences were observed between the M99 and the control strain (PS2161), as measured by CFU per gram of tissue within vegetations, kidneys, or spleens (data not shown). We also analyzed these findings by calculating a competition index, in which the ratio of M99 and PS2116 within tissues was normalized for the CFU of each strain within the inoculum. When analyzed by this approach, the densities of the GspB mutant strain PS2116 remained significantly reduced in all tissues, as compared with M99 (*P*<0.002), while the densities of the control strain were statistically similar.

**Table 6:** Effect of Sialyl-T antigen binding on virulence in a rat model of infective endocarditis.

Strain pairs	No. of animals	*S. gordonii* densities (Mean ± S.D. log_10_ CFU/g tissue)		
		Vegetations	Kidneys	Spleen
M99 (wt)		7.47±1.69	3.72±0.72	3.77±0.66
PS2116 (R484E)	11	6.81±1.70*	3.40±0.86*	3.20±0.72**
PS846 (Δ*gspB*)		5.76±2.15	2.72±1.32	3.32±1.10
PS2116 (R484E)	7	5.45±2.69	2.53±1.12	2.92±0.88

*p = 0.02;

**p = 0.001 compared with M99.

We then compared the relative virulence of PS2116 with PS846 (M99Δ*gspB*). Infective endocarditis was produced as above, using an inoculum containing the two strains in a 1∶1 ratio. When assessed at 72 h post-infection, PS2116 and PS846 had similar densities of organisms within all tissues (**Table 6**). Of note, the levels of both strains with vegetations were markedly lower than those achieved by wild-type M99 in the above studies. Thus, these two strains appeared comparably attenuated in the setting of endocarditis, indicating that platelet binding by the Siglec domain may be the predominant GspB interaction contributing to virulence in endocarditis.

### The binding regions of SRR adhesins have a modular organization

While overall sequence analysis of members of the SRR family has identified unifying sequence trends (**Fig. 1**), sequence comparisons of the binding regions have shown little homology. A structural comparison between the three distinct subdomains of GspB_BR_ (**Fig. 2A**) and the two distinct subdomains of the SRR adhesin Fap1 [43] immediately indicates that these family members adopt unrelated folds in their binding domains. Like GspB_BR_, Fap1_NR_ appears to be composed of independently-folded subdomains; however its binding region only contains two modules whereas the binding region of GspB_BR_ contains three. Intriguingly, Fap1_NR_ contains a helical subdomain at its N-terminus (Fap1_NR_-α) that does not resemble any of the subdomains of GspB_BR_, and a CnaA subdomain at its C-terminus (Fap1_NR_-β) that resembles the N-terminal CnaA subdomain of GspB_BR_ (GspB_BR-C_). This suggested to us that members of the SRR family could undergo reorganization of the modules within their host binding regions, with particular modules or combinations of modules conferring specific properties.

**Figure 9:**
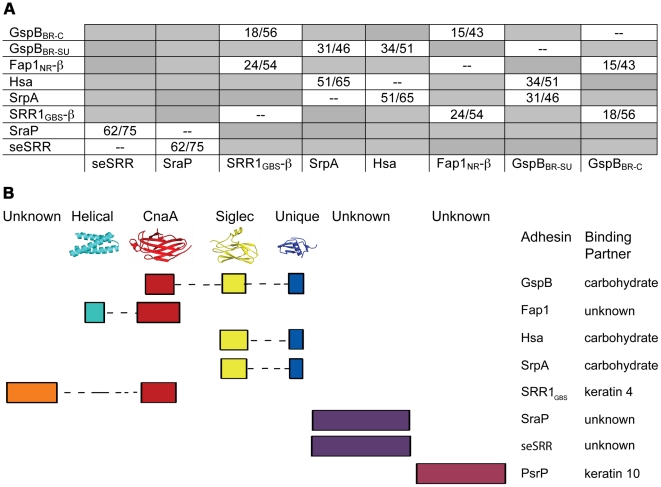
Modular organization of the binding regions within adhesins of the SRR superfamily. A. A summary of BLAST [50] or ClustalW [51] sequence alignments of the binding regions of SRR adhesins. The first number indicates sequence identity and the second indicates sequence similarity. GspB_BR_ has been divided into its subdomains, with GspB_BR-C_ indicating the CnaA subdomain and GspB_BR-SU_ indicating both the Siglec and Unique subdomains. Only the C-terminal subdomain of SRR1_GBS_ (SRR1_GBS_-β) and Fap1_NR_ (Fap1_NR_-β) are used in this analysis since the N-terminal domains do not share detectable sequence identity with other sequences used in this analysis. Boxes in grey indicate SRR pairs that do not have detectable sequence identity within the binding region. B. A schematic summarizing (A) The colored rectangles represent similar regions of sequence, and are drawn to scale. If known, representative structures of each domain are illustrated along the top, and binding partners are indicated along the side.

Accordingly, we re-analyzed the binding regions of selected members of the family using a new strategy, where we used BLAST [50] and ClustalW [51] to query the sequences of the binding regions of SRR adhesins with input sequences corresponding to subdomains of either GspB_BR_ or Fap1_NR_, or of short regions (∼200 amino acids) of sequences of structurally uncharacterized binding regions of SRR adhesins. These modified sequence comparisons strongly suggest that the binding regions of members of the SRR family have indeed evolved to contain modules (**Fig. 9**), and show several distinct groupings. For example, all three assessed SRR adhesins with carbohydrate binding partners contain the Siglec and Unique subdomains in tandem, strongly suggesting that the inclusion of these modules within the binding region confers lectin-like binding characteristics.

These focused sequence alignments additionally identify that a CnaA subdomain may be common in SRR adhesins, appearing in three of the eight binding regions that we assessed. As opposed to the combination of the Siglec and Unique subdomains, which are associated with carbohydrate binding, each adhesin containing a CnaA subdomain may have a different binding specificity (**Fig. 9**). Interestingly, in the SRR adhesins that were assessed, the CnaA subdomains were always found paired with sequence for another subdomain.

It is important to note that several of the binding regions of the SRR adhesins do not have significant sequence identity to any protein of known structure. For example, the binding region of *S. agalactiae* SRR1 (SRR1_GBS_) likely contains two subdomains, SRR1_GBS_-α at the N-terminus, which does not contain detectable sequence similarity to any protein of known fold, and SRR1_GBS_-β at the C-terminus, which has sequence similarity to MSCRAMMs and likely adopts a similar folding topology to CnaA. Likewise, no part of the binding regions of *S. pneumoniae* PsrP, *S. aureus* SraP, or *S. epidermidis* seSRR_BR_ exhibits detectable sequence similarity to any known protein, but the latter two have high sequence similarity to each other.

## Discussion

SRR adhesins have been identified in a variety of Gram-positive pathogens, and have been implicated as virulence factors in a wide spectrum of infections [5], [6], [12], [18]. The diversity of these infections (e.g., endocarditis, meningitis, pneumonia) and the broad scope of their anatomic locations are consistent with the binding regions of the SRR proteins differing considerably in their selectivity. Indeed, although only a few ligands for this family of proteins have been identified, they range from carbohydrates (such as sialyl-T antigen) [32] to proteins (such as keratins) [16], [17]. To date, however, the structural basis for this selectivity has been unknown.

In several members of the family (e.g. the SRR adhesins of *S. gordonii* and *S. pneumoniae*) the domain between the two SRR regions mediates binding. However, for many of these SRR adhesins (i.e. SraP of *S. aureus*), the host receptor has yet to be identified [6], [43], and the delineation of the binding region is assumed based upon sequence comparisons within the family. Among the best characterized of the SRR adhesins is GspB from *S. gordonii*, which has demonstrated binding affinity for the sialyl-T antigen carbohydrate decorating platelet glycoprotein GPIbα. Our crystal structure of the binding region, GspB_BR_, identified a modular organization with three subdomains (**Fig. 2A**), two of which are organized around Ig-folds. Proteins containing Ig-folds are commonly found within the mammalian immune system, where they exhibit a variety of functions; however, Ig-folds are not uncommon within pathogens, where they act exclusively as virulence factors. The first characterized bacterial protein to contain an Ig-fold was PapD, a chaperone for the assembly of pili in *E. coli* [37]. The Ig-fold has been observed in several other types of virulence factors, including components of pili (carbohydrate-binding fimbriae) [41], [52], and in adhesins of varying specificity for host receptors, including MSCRAMMs [34]–[36] and invasins [38]–[40].

Co-crystallization of GspB_BR_ with α-2,3-sialyl (1-thioethyl)galactose identified a specific binding site for this disaccharide within the Siglec subdomain (**Fig. 4B–D**), and the introduction of single point mutations within this region significantly reduced levels of binding of *S. gordonii* to glycocalicin. Moreover, the R484E substitution in GspB reduced platelet binding by M99 (**Fig. 7D** **,** **Fig. 8** **,** **Table 5**), and had a marked reduction in binding of the GST-GspB_BR_ fusion protein to sialyl-T antigen (**Fig. 7C**), confirming the importance of the Siglec subdomain for carbohydrate binding. These results indicate that this binding pocket within the Siglec subdomain is required for binding to sialyl-T antigen, and that the interaction of this GspB subdomain with sialyl-T antigen is important for binding to platelets.

When examined in a co-infection model of infective endocarditis, the isogenic variant of M99 expressing GspB-R484E (strain PS2116) was also significantly reduced in virulence (most notably a 78% reduction in bacterial levels within vegetations as compared with the parent strain). When PS2116 was tested with PS846 (M99Δ*gspB*) in this model, the strains had comparable but reduced densities within target tissues (as compared with WT M99), indicating that they were similarly reduced in virulence. These findings indicate that the predominant property of GspB contributing to the pathogenesis of endocarditis is its interaction with sialyl-T antigen. In previous *in vivo* studies, co-infection with M99 and PS846 yielded more pronounced differences between the strains, as measured by densities of organisms within target tissues [18]. This may reflect subtle experimental differences, such as inoculum size, but could also be due to residual binding activity of the mutated GspB. Of note, PS2116 had low but detectable levels of platelet binding *in vitro*. Similarly, the GST-GspB_BR_-R484E had measurable levels of binding to sialyl-T antigen, as compared to GST alone. This residual binding seen *in vitro*, presumably due to the other key residues identified by crystallography, may account for the residual virulence of the PS2116 strain. Alternatively, although ligands for GspB other than sialyl-T antigen have not been identified, it is conceivable that GspB has other interactions *in vivo* that contribute to virulence in this setting, beyond those mediating platelet binding.

Upon binding of the α-2,3-sialyl (1-thioethyl)galactose to GspB_BR_, a 40° interdomain angle straightening is observed between the CnaA and Siglec subdomains (**Fig. 6**, **Video S1**). The interdomain hinge is centered at the ion binding site, making the interdomain angle change reminiscent of calcium dependent interdomain angle changes observed in C-cadherin, which also contains modules of Ig-fold topology [53]. There are several possibilities for the origin and role of this interdomain reorganization in GspB_BR_. It is first possible that GspB_BR_ exhibits natural flexibility between these two domains, and that the difference in crystal contacts artifactually resulted in the straighter form of the protein being trapped in the co-structure of GspB_BR_ with α-2,3-sialyl (1-thioethyl)galactose. Intriguingly, however, an interdomain straightening is also observed in small angle x-ray scattering (SAXS) of the binding region of SRR protein Fap1_NR_ upon lowering the pH to 5.0, where Fap1_NR_ has highest affinity for its host receptor [43]. Those experiments, performed in solution, are not limited by crystal contacts and suggest that interdomain straightening upon ligand binding could be a conserved feature of members of the SRR family. Physiological roles of interdomain straightening upon ligand binding include improved hydrodynamics in the cardiovascular system or oral cavity. The elongation upon ligand binding is perhaps more compellingly reminiscent of the interdomain straightening that occurs upon the binding of P-selectin and β3-integrin to their respective ligands [54]. These proteins have been suggested to form “catch bonds” in order to have increased affinity to their ligand upon the application of tensile force. Each of these possibilities for the observed straightening of GspB_BR_ upon ligand binding is currently under investigation.

While both Siglecs and GspB_BR_ bind to carbohydrate receptors, the structural details of disaccharide binding to this novel bacterial Siglec bear little resemblance to the binding of sialic acid moieties to characterized mammalian Siglecs [44]–[46] (**Fig. 5**). Indeed, comparison of the binding sites reveals that the secondary structure of GspB_BR_ has a helix that is found at the same location where 3′ sialyllactose binds to Siglec-5 (**Fig. 5A**), thus eliminating the possibility of analogous carbohydrate binding. Instead, GspB_BR_ appears to use a β-grasp domain to form the specific host receptor binding site, a domain found in another group of sialic acid binding proteins, staphylococcal superantigen-like proteins (SSLs) that forms a V-like cleft for carbohydrate binding [47]–[49]. Nevertheless, both mammalian Siglecs and GspB_BR_ have binding pockets predominated by tyrosines and arginines. With a closer look at each binding site, in Siglecs, the sialic acid moiety of the binding partner makes a salt bridge with an arginine residue. This salt bridge is not present in the binding site of GspB_BR_ despite the presence of R484 and its key role in binding; this arginine instead binds to the C6 hydroxyl and the pyranose oxygen of the 1-thioethylgalactose (**Table 3**).

Importantly, the structure of GspB_BR_ provides insight into the binding repertoire of other SRR proteins, and specifically highlights which modules mediate binding to host carbohydrate receptors. Homologues of GspB containing regions with high sequence similarity to the Siglec and Unique subdomains (such as *S. gordonii* strain Challis Hsa and *S. sanguinis* SrpA) are also demonstrated lectins. Supporting this sequence analysis, our co-crystal structure of GspB_BR_ with α-2,3-sialyl (1-thioethyl)galactose demonstrates that the first two carbohydrates of host receptor bind within a pre-formed pocket on the Siglec subdomain. Using this experimental co-structure as a starting point, we could model binding of the sialyl-T antigen. This model is consistent with the third carbohydrate of this trisaccharide binding to an extended lobe on this pocket. This region of the binding pocket is still within the Siglec subdomain but extends toward the Unique subdomain (**Fig. S3**).

The structures of both GspB_BR_ and Fap1_NR_ identified a CnaA-like subdomain within the binding region, and our sequence analysis (**Fig. 9**) additionally predicts a CnaA subdomain at the C-terminus of the binding region of *S. agalactiae* SRR1 (SRR1_GBS_-β), an adhesin for keratin 4 [16]. As opposed to the Siglec subdomain, which contains an identifiable receptor binding site, the precise function of the CnaA subdomain is less clear. MSCRAMMs, such as CnaA from *S. aureus*, recognize peptides through the formation of a binding site between two Ig-like domains (**Fig. S4**). The bound peptide forms an additional strand that becomes a part of the Ig-fold. In contrast, GspB_BR_, Fap1_NR_, and SRR1_GBS_ each apparently contain a single CnaA subdomain amongst the modules in the binding region (**Fig. 9**). Our modeling suggests that binding of a peptide between two domains in a manner analogous to binding of a peptide to other MSCRAMMs is not feasible. Indeed, an additional strand found in the subdomain of GspB_BR_ occupies the peptide binding site of MSCRAMMs. (**Fig. S4,** **Fig. 3C** **strand A′**). Nevertheless, in SRR adhesins, the CnaA subdomain is always found together with at least one other subdomain, suggesting that the function may either require or be tuned by the presence of a second subdomain. Indeed, recent studies on the binding region of Fap1 support this hypothesis. The structures of Fap1_NR_-α (helical subdomain) and Fap1_NR_-β (CnaA subdomain) identified a region of surface-exposed hydrophobic residues on each domain that is predicted to be contiguous based upon SAXS. Fap1 normally mediates binding of this bacterium to the oral cavity, and mutagenesis of these hydrophobic residues abrogated binding to an *in vitro* tooth model consisting of saliva-coated hydroxylapatite [43].

Our sequence analysis additionally predicts that the binding regions of other SRR adhesins contain modules that are structurally distinct from those identified in either GspB_BR_ or Fap1_NR_. Two of these, *S. aureus* SraP_BR_ and *S. epidermidis* seSRR_BR_, have high sequence identity to each other, but no detectable sequence similarity to any other SRR adhesin (**Fig. 9**). This strongly suggests that they bind a common, as yet unidentified, host receptor that is distinct from the carbohydrates and keratins recognized by other SRR adhesin family members. By comparison, neither *S. pneumoniae* PsrP_BR_, which has been demonstrated to bind keratin 10, nor the N-terminal subdomain of *S. agalactiae* SRR1_BR_, which binds keratin 4, have detectable sequence similarity to any currently available sequence (**Fig. 9**). Given this analysis, it is clear that a more detailed understanding of the binding characteristics of the SRR adhesin family will require that for each module type, the binding partner should be identified, and the structure of the binding region should be determined both alone and in complex with the appropriate host receptor.

## Materials and Methods

### Ethics statement

All procedures involving rats were approved by the Los Angeles Biomedical Research Institute animal use and care committee, following the National Institutes of Health guidelines for animal housing and care. Platelets were collected using a protocol approved by the UCSF Committee on Human Research (H1193-25513-07) and by the VUMC Human Research Protection Program IRB Committee (110364).

### Protein expression and purification

The DNA encoding residues 233–617 of GspB (GspB_BR_) was cloned into the pGEX vector containing an N-terminal glutathione S-transferase (GST) fusion tag as described [11]. This clone contains a single base pair change as compared to the deposited NCBI sequence that results in a serine at position 444 instead of an asparagine. The protein was expressed with *E. coli* BL21 Gold (Stratagene) in Luria Broth Medium as described [55] and purified using a GST affinity column (GE Healthcare). The GST tag was removed from GspB_BR_ using Factor Xa, and GspB_BR_ was further purified using size exclusion chromatography on a Superdex 200 10/300 GL column (GE Healthcare) with buffer containing 20 mM Tris pH 7.4 [55].

### Crystallization

All crystals of GspB_BR_ were grown using the hanging drop vapor diffusion technique at 23°C [55] using 1 µl protein solution and 1 µl of reservoir solution equilibrated against 1 ml of the reservoir solution. Crystals of native GspB_BR_ grew from two chemically distinct sets of conditions. The first set of conditions included GspB_BR_ at a concentration of 6 mg/ml buffered in 20 mM Tris pH 7.4 and equilibrated against a reservoir solution containing 33% Jeffamine ED-2001, 0.1 M HEPES pH 7.5 and 0.15 M KCl at 23°C. These crystals belonged to the primitive orthorhombic space group *P*2_1_2_1_2_1_ with unit cell dimensions a = 33.7 Å, b = 96.8 Å, c = 100.2 Å with α = β = γ = 90°. Prior to data collection, crystals were cryo-protected in a solution containing all of the chemical components of each reservoir solution and 15% glycerol then flash-cooled in liquid nitrogen. The dataset used for refinement merged to 1.4 Å resolution.

The second type of native GspB_BR_ crystals grew when the protein was equilibrated against a reservoir solution containing 25% polyethylene glycol 3350, 0.1 M HEPES pH 7.5, 0.15 M NH_4_CH_3_COO, and 10 mM spermidine and induced crystal growth from 10 mg/ml GspB_BR_ buffered in 20 mM HEPES pH 7.5 at 18°C. These crystals also belonged to the orthorhombic space group *P*2_1_2_1_2_1_, but had altered unit cell dimensions of a = 33.4 Å, b = 86.7 Å, c = 117.9 Å, α = β = γ = 90°. Prior to data collection, these crystals were flash cooled in liquid nitrogen without additional cryo-protectant. The best dataset from this crystal form merged to 2.0 Å resolution.

Crystals of GspB_BR_ in complex with the α-2,3-sialyl (1-thioethyl)galactose disaccharide were grown using the hanging drop vapor diffusion method with 6 mg/mL GspB_BR_ in buffer containing 1 mM α-2,3-sialyl (1-thioethyl)galactose and 20 mM Tris pH 7.4. The reservoir solution contained 8% PEG 3350, 7.5 mM CoCl_2_, 7.5 mM NiCl_2_, 7.5 mM CdCl_2_, 7.5 mM MgCl_2_, and 0.1 M HEPES pH 7.5. Crystals grew within two days and were cryo-protected in reservoir solution supplemented with 20% glycerol and flash-cooled in liquid nitrogen. The disaccharide co-crystals formed in the primitive monoclinic space group *P*2_1_ with unit cell dimensions a = 69.9 Å, b = 34.0 Å, c = 83.4 Å with α = γ = 90° and β = 99.2°. The best data from this crystal form merged to 1.9 Å resolution.

### Data collection and processing

Crystals were assessed for diffraction quality at the Stanford Synchrotron Radiation Lightsource (SSRL) beamlines 9-2, 11-1, and 12-2 and the Life Sciences Collaborative Access Team (LS-CAT) beamlines ID-21-D/F/G. Datasets were collected using the beamlines, temperatures, wavelengths, and detectors listed in **Table 1** and **Table 2**. All data were processed using the HKL2000 [56] and CCP4 [57] suites of programs.

### Preparation of heavy atom derivatives and structure determination

A Dy^3+^ derivative was prepared by soaking pre-formed crystals of GspB_BR_ in 1 mM DyCl_3_ for three days. Data were collected at three wavelengths near the Dy^3+^ L3 edge (Table 2). Dy^3+^ bound to a single site in the protein as determined using the SHELXD [58] subroutine in the program SHARP [59]. While HoCl_3_ also successfully derivatized GspB_BR_, non-isomorphism between crystals prevented the use of this second derivative in a traditional MIR calculation. The non-isomorphism was so severe that phases calculated in SHARP [59] only used data from a single DyCl_3_ soaked crystal and did not include a native dataset for reference. This process resulted in reasonable phasing statistics and an overall figure of merit of 0.83 at 2.0 Å resolution (**Table 2**). Phases were improved by solvent flattening in DM [60] which produced electron density maps of high quality (**Fig. 2B**). Automated chain tracing was performed using PHENIX [61], which was able to trace residues 251–316 and 327–601, representing 94.7% of the model. This resulted in an initial R_cryst_ of 23.8% and R_free_ of 25.5%.

The lack of isomorphism between crystals prevented transfer of these initial coordinates to other data sets using a simple rigid body refinement. As a result, the model from the Dy^3+^ data set was transferred to the remaining data sets using the program PHASER [62] followed by rigid body refinement in CNS [63]. Each structure was subjected to alternate rounds of model building using the program COOT [64] and refinement using CNS [63] and REFMAC [65].

The coordinates for the α-2,3-sialyl (1-thioethyl)galactose were built using CCP4i Sketcher [66] and PRODRG [67]. Refinement statistics for all final models are listed in **Table 1**. Figures were created using PyMOL [68], and inter-domain rotations were determined using DynDom [69].

### Synthesis of the α-2,3-sialyl (1-thioethyl)galactose disaccharide

Based upon work by Danifshesky and coworkers [70], we developed a four-step synthesis for the sialyl-T antigen precursor, α-2,3-sialyl (1-thioethyl)galactose (see **Supporting Protocol S1**). The correct synthesis of the disaccharide was verified by NMR (**Fig. S2**). The α-2,3-sialyl (1-thioethyl)galactose was resuspended in water for all applications.

### Site-directed Mutagenesis

Point mutations of *gspB* were introduced into the *S. gordonii* chromosome via a strategy that involved recombination by double cross-over between *gspB* codons 487–602 and a gene approximately 300 bp upstream. This approach ensured incorporation of only the intended mutation of *gspB* codons ranging from 399 to 485, and avoided possible imprecise recombination within the SRR regions. As a first step, the 5′ end of *gspB* (codons 1 to 486 along with 200 nts from a non-coding region upstream) was replaced with a chloramphenicol resistance cassette as follows. A 0.5 kb segment of a gene of unknown function upstream of *gspB* was amplified using PCR. The product was digested with XhoI and ClaI and then cloned upstream of the *cat* gene in pC326 [71]. A segment spanning *gspB* codons 487 to 602 was then amplified using primers B487F and B602R, digested with SpeI and NotI, and cloned downstream of the *cat* gene. The resulting plasmid, pC326Δ5′B, was used to transform *S. gordonii* strain M99 as described [4]. One of the chloramphenicol-resistant transformants, (M99 Δ5′*gspB::cat*), was selected for subsequent gene replacement.

A series of plasmids was then constructed to facilitate the replacement of the 5′ end of *gspB* in M99 Δ5′*gspB::cat*. The XhoI-ClaI fragment from pC326Δ5′B was cloned upstream of the *spec* gene in pS326 [72], and a 1 kb NsiI-SpeI fragment of *gspB* (spanning codons 1 to 296), was cloned downstream. A SpeI-NotI fragment spanning codons 296 to 602 was then cloned downstream of the NsiI-SpeI fragment. Point mutations in the resulting plasmid, pS326B602, were generated by a two-stage PCR procedure. In the first stage, primer 25F along with a reverse *gspB* primer, or the corresponding *gspB* forward primer along with primer B602R, were used to amplify the upstream or downstream segments, respectively. The two PCR products were combined for the second stage and then amplified using primers 25F and B602R. The PCR product was digested with SpeI and NotI and then used to replace the corresponding segment of pS326B602. The incorporation of only the intended change in any segment generated by PCR was confirmed by DNA sequence analysis. Plasmids were then used to transform M99 Δ5′*gspB::cat*, resulting in a replacement of *Δ*5′*gspB::cat* with a *5′gspB::spec* variant. As a control, the wild-type *gspB* sequence along with the *spec* cassette (pS326B602) was also crossed into the M99 Δ5′*gspB::cat* chromosome (generating strain PS2161). Transformants were selected on spectinomycin and scored for the loss of chloramphenicol resistance. Expression of the variant GspB proteins on the bacterial cell surface was verified by western blotting as described [72].

### GspB_BR_ binding to biotinylated sialyl-T antigen

GST, GST-GspB_BR_ and GST-GspB_BR_-R484E were purified from *E. coli* as described [55]. The purified proteins were diluted to 320 µM into DPBS, serial two-fold dilutions were made, and 50 µl of each dilution was added to wells of a 96-well microtiter plate. After incubating the plate overnight at 4°C, unbound proteins were removed by aspiration and wells were rinsed with 100 µl DPBS. Biotinylated sialyl-T antigen (sialyl-T antigen conjugated to biotin via a polyacrylamide linker; GlycoTech Corporation) was diluted to 50 µg/ml in DPBS containing 1× Blocking Reagent (Roche), 50 µl was added to each well, and the plate was incubated for 2 h at RT with vigorous rocking. After removing unbound biotin-sialyl-T antigen, wells were rinsed three times with 100 µl DPBS, 50 µl of streptavidin-conjugated horseradish peroxidase (0.1 µg/ml in DPBS) was added to each well and the plate was incubated for 1 h at 23°C. The wells were washed twice with 100 µl DPBS, and then 200 µl of a solution of OPD (0.4 mg/ml citrate-phosphate buffer) was added to each well. The contents of the wells were mixed by gently vortexing the plate, and the absorbance at 450 nm was measured 20 min after the addition of the OPD substrate. Data were plotted as the means ± standard deviations, with *n* = 3.

### Binding of *S. gordonii* to glycocalicin and platelet monolayers

The binding of *S. gordonii* to immobilized glycocalicin was performed as described previously [32]. In brief, strains were grown for 18 hr, washed twice with DPBS, sonicated briefly to disrupt aggregated cells, and then diluted to approximately 2×10^7^ per ml. To determine whether α-2,3 sialyl (1-thioethyl)galactose inhibited binding to glycocalicin, the washed and sonicated bacteria were diluted into DPBS or DPBS containing 44 mM α-2,3-sialyl (1-thioethyl)galactose, pH 7.5. The bacterial suspensions were then applied to wells of a microtiter plate that had been coated with glycocalicin (1.25 µg/well). After 2 h at room temperature, the unbound bacteria were removed by aspiration. Wells were washed three times with DPBS, and the bound bacteria were released by trypsinization. The number of input and bound bacteria were determined by plating serial dilutions of the bacterial suspensions on sheep blood agar plates, and binding was expressed as the percent of the input bound to glycocalicin. The binding of *S. gordonii* to immobilized human platelets was assessed as described previously [4]. Results of both assays are reported as the means ± standard deviations, with n = 6. Differences in binding were compared by the unpaired *t*-test.

### DIC and fluorescence microscopy

*S. gordonii* strains M99 wild type, PS2116, and PS846 were grown in 5 mL of Todd Hewitt Broth at 37°C without shaking for 18 hours. Cells were then vortexed to resuspend bacteria and spun down at 4000× g for ten minutes. The supernatant was removed and the bacteria washed twice with 5 mL of DPBS containing MgCl_2_ and CaCl_2_. The bacteria were then resuspended in 5 mL of DPBS and sonicated briefly to disrupt aggregated clumps. To 1 mL of each cell suspension, 500 nM 4′,6-diamidino-2-phenylindole (DAPI) was added.

Platelets were fixed using 1.6% paraformaldehyde. 500 µL of platelets were mounted on poly-L-lysine coated cover slips (in a 6-well tray) which was spun at 400× g for ten minutes in order to promote platelet adherence to the cover slips. Excess platelets were removed by washing with Tris Buffered Saline (TBS) and 1 mL of TBS was added to each well. 500 µL of the bacterial suspension was added to the platelets and the samples were rocked vigorously for 30 minutes at 23°C. Excess bacteria were then removed by washing three times with TBS. Each sample was mounted onto a slide for microscopy. Slides were imaged using Nikon TiE Inverted light microscope equipped with a Photometrics CoolSnap HQ CCD camera. Platelets were imaged with a 100×1.49NA objective using DIC optics and a DAPI filter cube. Image J software was used to create the contrast and composite images.

### Rat model of infective endocarditis

A competition model of infective endocarditis was produced in Sprague–Dawley female rats (250–300 g) as described previously [18]. In brief, the animals were anesthetized with ketamine (35 mg/kg) and xylazine (10 mg/kg). A sterile polyethylene catheter was surgically placed across the aortic valve of each animal, such that the tip was positioned in the left ventricle. Catheters were left in place throughout the study. Catheterized animals were then infected intravenously (IV) with an inoculum containing 2×10^5^ CFU of both strains (i.e., a 1∶1 mixture of i) *S. gordonii* strain M99 and strain PS2116 (5′*gspB^R484E^::spec*), ii) M99 and strain PS2161 (5′*gspB::spec* control strain), or iii) PS2116 and strain PS846 (M99 Δ*gspB*::pEVP3; Cm^R^) [18], [73]. At 72 h post-infection, animals were sacrificed with thiopental (100 mg, intraperitoneally). Animals were included in the final analysis only if the catheters were correctly positioned across the aortic valve at the time of sacrifice, and if macroscopic vegetations were seen. All cardiac vegetations, as well as samples of the kidneys and spleens were harvested, weighed, homogenized in saline, serially diluted, and plated onto Todd Hewitt agar (THA) (for the parental *S. gordonii* strain M99) and THA containing 100 µg/ml of spectinomycin (for strains PS2116 and PS2161) or chloramphenicol 5 mg/ml (for strain PS846) for quantitative culture, to determine the number of CFU/g of *S. gordonii* strains within tissues. After 48 h of incubation at 37°C, bacterial colonies were counted. The number of bacteria within tissues was expressed as the log_10_ CFU per gram of tissue. Differences between means were compared for statistical significance by the paired *t*-test, using *p*≤0.05 as the threshold for significance. The data were also analyzed by calculating a “competition index,” which was defined as the ratio of *S. gordonii* strain M99 and strain PS2116 or PS2161, as well as PS2116 and PS846, within tissues for each animal, normalized by the ratio of organisms in the inoculum. The mean of the log_10_ normalized ratios was tested against the hypothesized ‘no effect’ mean value of 0, as described previously, using a paired *t*-test.

### Accession numbers

The coordinates and structure factors for *S. gordonii* strain M99 GspB_BR_ have been deposited in the Research Collaboratory for Structural Bioinformatics Protein Data Bank with accession codes 3QC5 (native 1), 3QC6 (native 2, crystal form 2), and 3QD1 (α-2,3-sialyl (1-thioethyl)galactose bound).

## Supporting Information

Figure S1 The cation binding site. Stereo view of the cation binding site superpositioned with 2m|F_o_|−d|F_c_| electron density calculated in REFMAC5 [65] and contoured to 1.5 σ. Carbons are shown in *grey*, oxygens are shown in *red*, nitrogens are shown in *blue* and the cation is shown as a *purple* sphere. Bond distances are shown in Å.
(TIF)

Figure S2 ^1^H NMR, 400 MHz (CDCl_3_) spectrum of α-2,3-sialyl (1-thioethyl)galactose.
(TIF)

Figure S3 Predicted binding of sialyl-T antigen to the receptor binding pocket. Using the co-structure of GspB_BR_ with α-2,3-sialyl (1-thioethyl)galactose as a starting point, we developed a model for binding of sialyl-T antigen to the Siglec subdomain. Coordinates for sialyl-T antigen were prepared in COOT [64] by manually linking the 3-position of N-acetylgalactosamine to the 1-position of the galactose in α-2,3-sialyl (1-thioethyl) galactose and removing the thioethyl group. The position of the α-2,3-sialyl (1-thioethyl) galactose group was fixed to its position in the experimentally-determined structure and the galactosamine manually optimized to avoid steric clashes. In this model, all three sugars bind within a contiguous pre-formed pocket on the surface of the protein between the Siglec and Unique subdomains. The Siglec subdomain is colored *yellow*, the Unique subdomain is colored *blue*, and the modeled sialyl-T antigen is colored with *gray* carbons.
(TIF)

Figure S4 Binding of MSCRAMMs to fibrinogen receptors. The interdomain peptide binding observed in SdrG (PDB entry 1R17) uses a “dock, lock, and latch” mechanism of fibrinogen recognition [34] is unlikely to be used by SRR adhesins. This interdomain mechanism requires two adjacent Ig-fold domains that create a binding site; the fibrinogen peptide binds between these two domains. Modeling additional interdomain angles in GspB_BR_ suggests that the short linker region between the CnaA and Siglec subdomains will prevent the interdomain angle from becoming sufficiently acute to allow the Siglec subdomain to act as a surrogate second subdomain. In addition, GspB_BR_ contains an additional strand at the N-terminus of the CnaA subdomain (**Fig. 3C** **strand A'**) that occupies the peptide binding site of MSCRAMMs. Unbound SdrG is colored *gray* and bound SdrG is colored *red* with the fibrinogen peptide colored *cyan*.
(TIF)

Video S1 Range of interdomain angles observed in crystals of GspB_BR_. The movie morphs between states observed in the crystal structures and was produced using LSQMAN [75] and PyMOL [68]. The most extended conformation was observed in the co-structure of GspB_BR_ with the α-2,3-sialyl (1-thioethyl)galactose disaccharide.
(MP4)

Test S1 Results and discussion regarding the cation binding site.
(DOCX)

Protocol S1 Identification of the cation binding site and synthesis of α-2,3-sialyl (1-thioethyl)galactose.
(DOC)
