# Correction: A Structural Model for Binding of the Serine-Rich Repeat Adhesin GspB to Host Carbohydrate Receptors

**DOI:** 10.1371/annotation/9bb4b6f9-d220-4c81-bdbc-ebdb33e6d892

**Published:** 2012-12-17

**Authors:** Tasia M. Pyburn, Barbara A. Bensing, Yan Q. Xiong, Bruce J. Melancon, Thomas M. Tomasiak, Nicholas J. Ward, Victoria Yankovskaya, Kevin M. Oliver, Gary Cecchini, Gary A. Sulikowski, Matthew J. Tyska, Paul M. Sullam, T. M. Iverson

In the original coordinates (PDB entry 3QD1) for the costructure between the glycan and GspB, the occupancy of the disaccharide was set to 0.15, and very close contacts between the glycan and GspB are observed. While additional electron density only appeared in this location when GspB was crystallized in the presence of this glycan, the authors would like to clarify that these crystallographic data only suggest a possible gross location of the glycan-binding site and do not explicitly reveal the precise position of all atoms of the glycan or the atomic details of the receptor-GspB interaction. As a result, the receptor-GspB distances highlighted in Figures 4B and 5B may be incorrect. The PDB has accordingly been modified (new PDB entry 4I8E) with the occupancy of the glycan set to 0.0. This was done to provide a marker for the location of the electron density that appeared when the glycan was included in crystallization. In consideration of other evidence presented in the manuscript, the authors do not alter the conclusion that this site appears to be of functional importance for the pathogen-host interaction. The editors have reviewed and agreed with this correction.

