# Supplementary figures and images for: A Structural Model for Binding of the Serine-Rich Repeat Adhesin GspB to Host Carbohydrate Receptors
Source: PLoS Pathog. 2011 Jul 7;7(7):e1002112. doi: 10.1371/journal.ppat.1002112 (PMC3131266; doi:10.1371/journal.ppat.1002112)

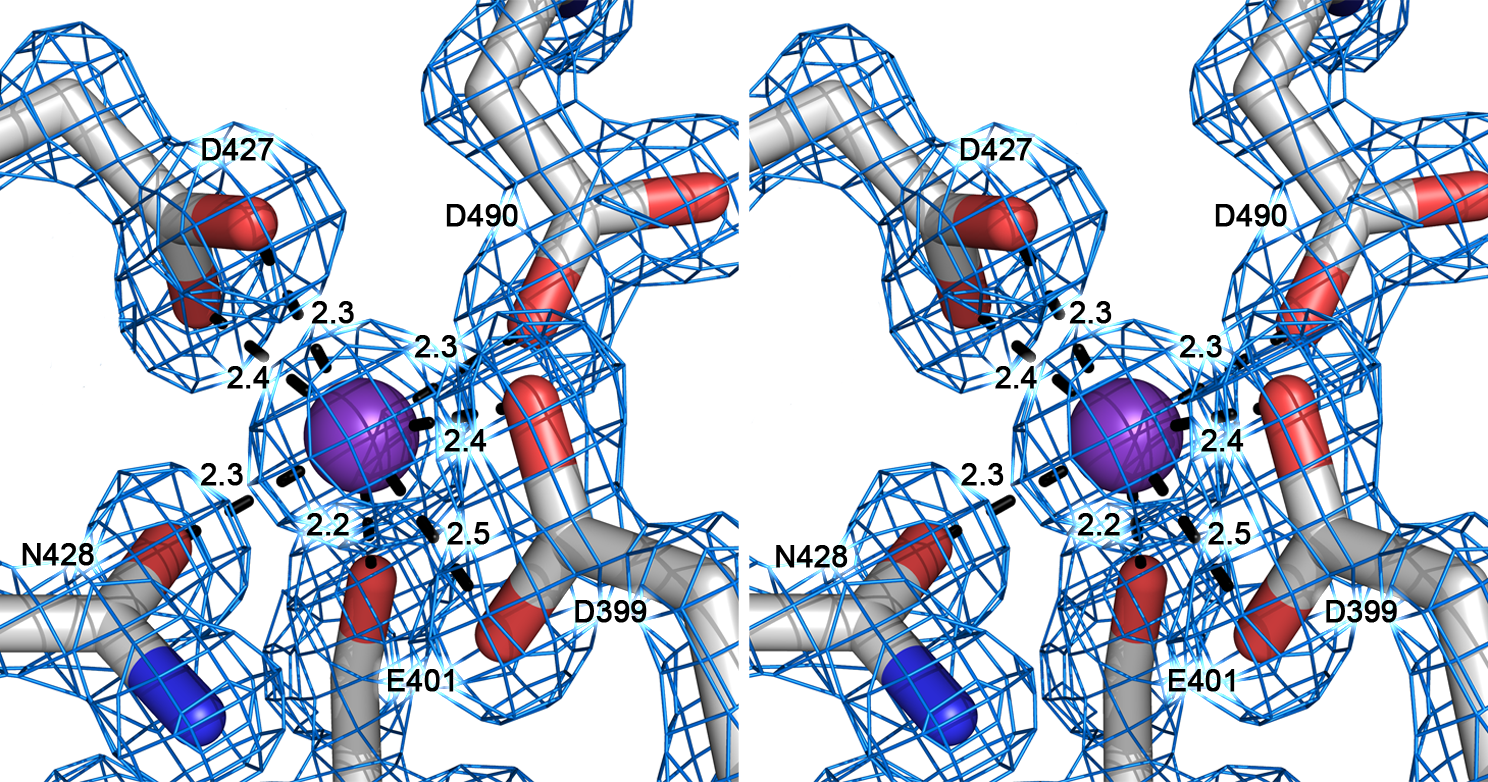

Supplement: Figure S1 — The cation binding site. Stereo view of the cation binding site superpositioned with 2m|Fo|−d|Fc| electron density calculated in REFMAC5 [65] and contoured to 1.5 σ. Carbons are shown in grey, oxygens are shown in red, nitrogens are shown in blue and the cation is shown as a purple sphere. Bond distances are shown in Å. (TIF) [file ppat.1002112.s001.tif]

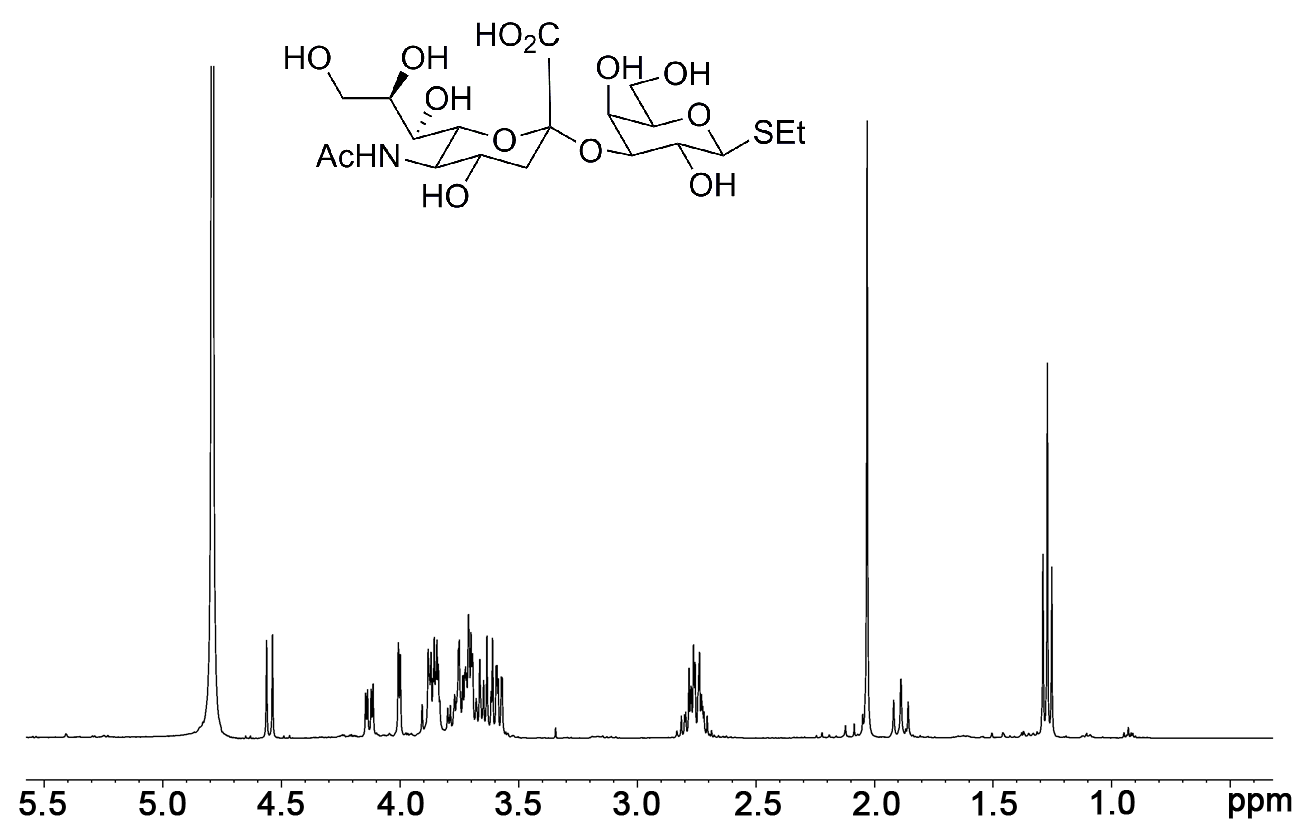

Supplement: Figure S2 — 1H NMR, 400 MHz (CDCl3) spectrum of α-2,3-sialyl (1-thioethyl)galactose. (TIF) [file ppat.1002112.s002.tif]

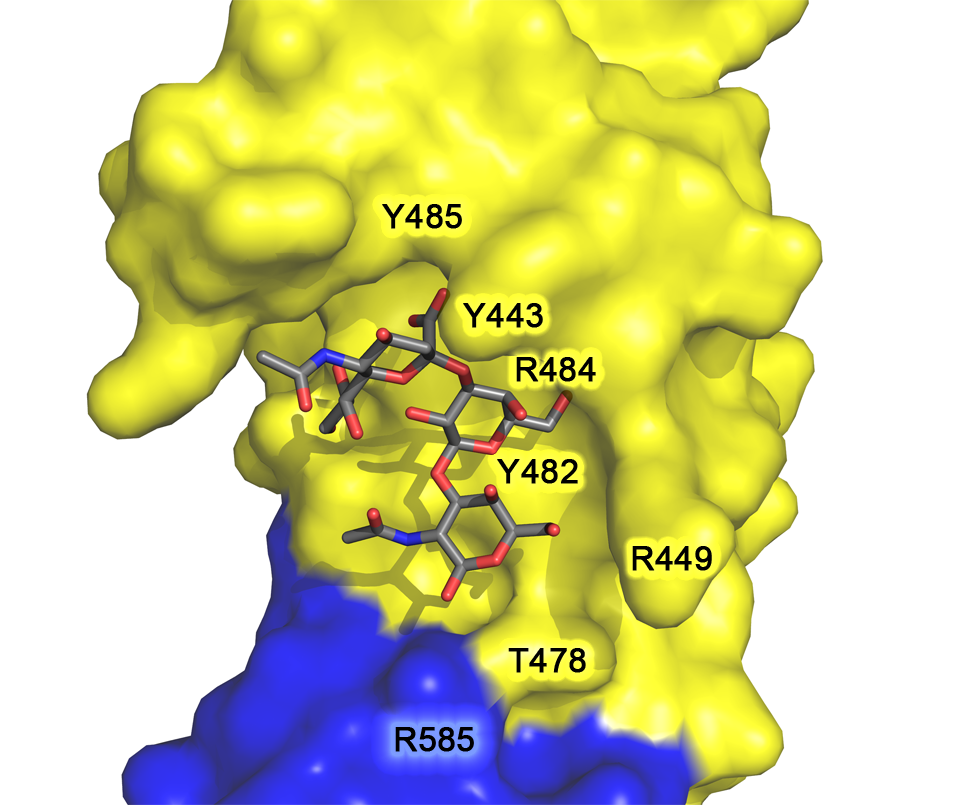

Supplement: Figure S3 — Predicted binding of sialyl-T antigen to the receptor binding pocket. Using the co-structure of GspBBR with α-2,3-sialyl (1-thioethyl)galactose as a starting point, we developed a model for binding of sialyl-T antigen to the Siglec subdomain. Coordinates for sialyl-T antigen were prepared in COOT [64] by manually linking the 3-position of N-acetylgalactosamine to the 1-position of the galactose in α-2,3-sialyl (1-thioethyl) galactose and removing the thioethyl group. The position of the α-2,3-sialyl (1-thioethyl) galactose group was fixed to its position in the experimentally-determined structure and the galactosamine manually optimized to avoid steric clashes. In this model, all three sugars bind within a contiguous pre-formed pocket on the surface of the protein between the Siglec and Unique subdomains. The Siglec subdomain is colored yellow, the Unique subdomain is colored blue, and the modeled sialyl-T antigen is colored with gray carbons. (TIF) [file ppat.1002112.s003.tif]

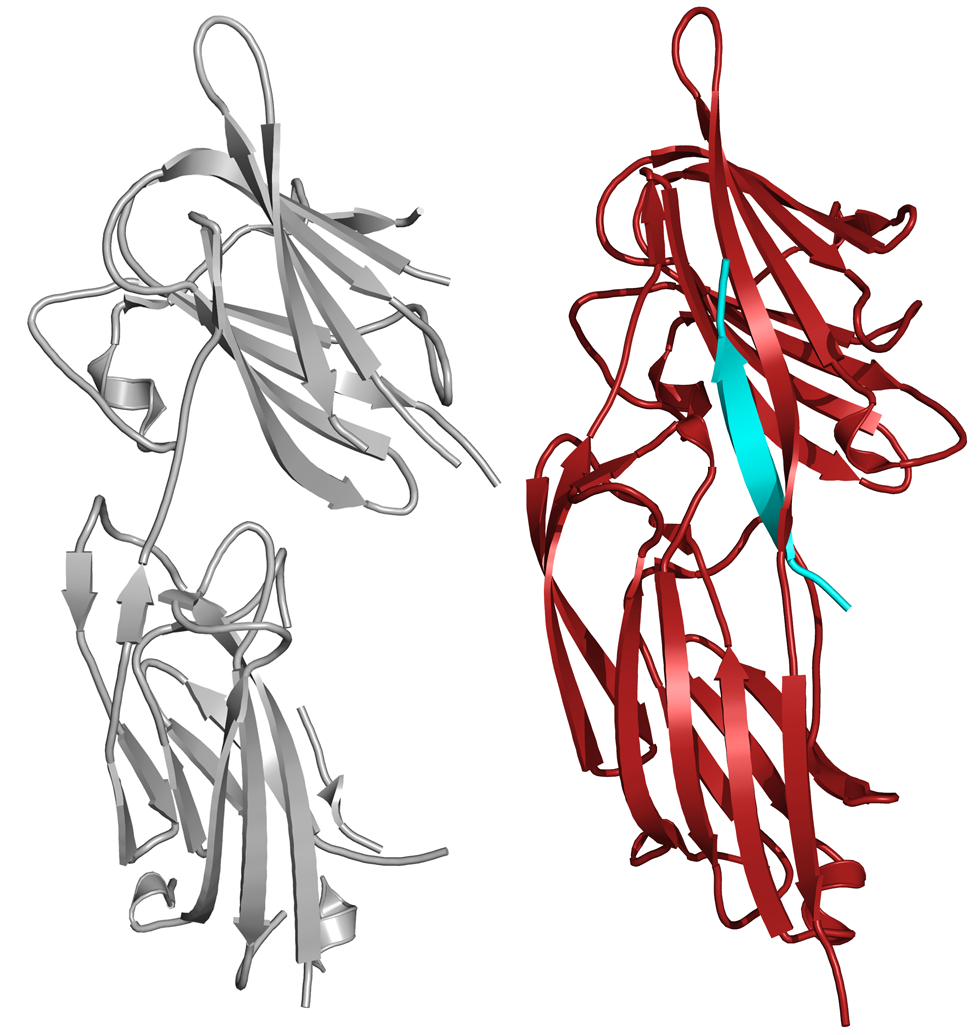

Supplement: Figure S4 — Binding of MSCRAMMs to fibrinogen receptors. The interdomain peptide binding observed in SdrG (PDB entry 1R17) uses a “dock, lock, and latch” mechanism of fibrinogen recognition [34] is unlikely to be used by SRR adhesins. This interdomain mechanism requires two adjacent Ig-fold domains that create a binding site; the fibrinogen peptide binds between these two domains. Modeling additional interdomain angles in GspBBR suggests that the short linker region between the CnaA and Siglec subdomains will prevent the interdomain angle from becoming sufficiently acute to allow the Siglec subdomain to act as a surrogate second subdomain. In addition, GspBBR contains an additional strand at the N-terminus of the CnaA subdomain ( Fig. 3C strand A') that occupies the peptide binding site of MSCRAMMs. Unbound SdrG is colored gray and bound SdrG is colored red with the fibrinogen peptide colored cyan. (TIF) [file ppat.1002112.s004.tif]
