# Supplementary material for: A Structural Model for Binding of the Serine-Rich Repeat Adhesin GspB to Host Carbohydrate Receptors
Source: PLoS Pathog. 2011 Jul 7;7(7):e1002112. doi: 10.1371/journal.ppat.1002112 (PMC3131266; doi:10.1371/journal.ppat.1002112)
Supplement: Test S1 — Results and discussion regarding the cation binding site. (DOCX) [file ppat.1002112.s006.docx]

**A Structural Model for Binding of the Serine-Rich Repeat Adhesin GspB to Host Carbohydrate Receptors**

**Tasia M. Pyburn^1,2^, Barbara A. Bensing^3^, Yan Q. Xiong^4^, Bruce J. Melancon^2,5,$^, Thomas M. Tomasiak^1,2,%^, Nicholas J. Ward^1^, Victoria Yankovskaya^6^, Kevin M. Oliver^2,5^, Gary Cecchini^6,7^, Gary A. Sulikowski^2,5^, Matthew J. Tyska^8^, Paul M. Sullam^3^, and T. M. Iverson^1,2,9,*^**

**Supporting Text S1**

**^1^**Departments of Pharmacology, ^8^Cell and Developmental Biology, and ^9^Biochemistry, Vanderbilt University Medical Center, Nashville, Tennessee, United States of America

^2^Vanderbilt Institute of Chemical Biology, Nashville, Tennessee, United States of America

**^3^**Department of Medicine, Veterans Affairs Medical Center, University of California, San Francisco, California, United States of America

^4^Department of Medicine, Harbor-UCLA Medical Center, Torrance, California, United States of America

^5^Department of Chemistry, Vanderbilt University, Nashville, Tennessee, United States of America, and

^6^Molecular Biology Division, Veterans Affairs Medical Center, San Francisco, California, United States of America

^7^Department of Biochemistry & Biophysics University

Running Head: Carbohydrate recognition by GspB

^$^Present address: Vanderbilt Program in Drug Discovery, Department of Pharmacology, Nashville, Tennessee, United States of America

^%^Present address: Molecular Structure Group, University of California, San Francisco, California, United States of America

*To whom correspondence should be addressed. Email: [tina.iverson@vanderbilt.edu](mailto:tina.iverson@vanderbilt.edu)**SUPPORTING TEXT**

**Cation Binding site**

The bond distances and seven-coordination number of the cation binding site identified within the Siglec subdomain of GspB_BR_ (**Fig. S1**) suggest that Ca^2+^ should bind preferentially. However, crystallization of GspB_BR_ in the presence of a variety of cations (K^+^, Cd^2+^, Ho^3+^, Dy^3+^) resulted in spontaneous incorporation of each within this site (see **Supporting Protocols S1** for a detailed description of the assignment of ions in each situation), suggesting that cation binding at this location is both labile and promiscuous. To evaluate if the cation identity affects the geometry of the metal binding site, we chelated as-isolated GspB_BR_, homogeneously incorporated the site with Ca^2+^, and determined the structure (not shown). When compared to crystal structures of GspB_BR_ containing K^+^, Cd^2+^, Dy^3+^, or Ho^3+^ at this site, no significant structural differences were observed.
