# Supplementary material for: A Structural Model for Binding of the Serine-Rich Repeat Adhesin GspB to Host Carbohydrate Receptors
Source: PLoS Pathog. 2011 Jul 7;7(7):e1002112. doi: 10.1371/journal.ppat.1002112 (PMC3131266; doi:10.1371/journal.ppat.1002112)
Supplement: Protocol S1 — Identification of the cation binding site and synthesis of α-2,3-sialyl (1-thioethyl)galactose. (DOC) [file ppat.1002112.s007.doc]

**A Structural Model for Binding of the Serine-Rich Repeat Adhesin GspB to Host Carbohydrate Receptors**

**Tasia M. Pyburn^1,2^, Barbara A. Bensing^3^, Yan Q. Xiong^4^, Bruce J. Melancon^2,5,$^, Thomas M. Tomasiak^1,2,%^, Nicholas J. Ward^1^, Victoria Yankovskaya^6^, Kevin M. Oliver^2,5^, Gary Cecchini^6,7^, Gary A. Sulikowski^2,5^, Matthew J. Tyska^8^, Paul M. Sullam^3^, and T. M. Iverson^1,2,9,*^**

**Supporting Protocol S1**

**^1^**Departments of Pharmacology, ^8^Cell and Developmental Biology, and ^9^Biochemistry, Vanderbilt University Medical Center, Nashville, Tennessee, United States of America

^2^Vanderbilt Institute of Chemical Biology, Nashville, Tennessee, United States of America

**^3^**Department of Medicine, Veterans Affairs Medical Center, University of California, San Francisco, California, United States of America

^4^Department of Medicine, Harbor-UCLA Medical Center, Torrance, California, United States of America

^5^Department of Chemistry, Vanderbilt University, Nashville, Tennessee, United States of America, and

^6^Molecular Biology Division, Veterans Affairs Medical Center, San Francisco, California, United States of America

^7^Department of Biochemistry & Biophysics University

Running Head: Carbohydrate recognition by GspB

^$^Present address: Vanderbilt Program in Drug Discovery, Department of Pharmacology, Nashville, Tennessee, United States of America

%Present address: Molecular Structure Group, University of California, San Francisco, California, United States of America

*To whom correspondence should be addressed. Email: [tina.iverson@vanderbilt.edu](mailto:tina.iverson@vanderbilt.edu)

**SUPPORTING PROTOCOL**

**Assignment of metals to the cation binding site**

While the seven-coordinate nature of the cation binding site certainly suggests that Ca^2+^ should bind preferentially, in the crystal structure of as-isolated GspB_BR_, significant |F_o_| - |F_c_| difference electron density is observed when Ca^2+^ is modeled at this site. This could be a result of misincorporation of the cations in heterologously expressed protein; however, the crystallization conditions contained 150 mM K^+^, and emission spectra of fully formed crystals revealed K^+^. This instead suggests that K^+^ spontaneously replaced the cation at this site. Corroborating that interpretation, derivatization of GspB_BR_ crystals with 10 mM of either Dy^3+^ or Ho^3+^ resulted in the heavy atom binding at this location with nearly 100% occupancy, suggesting that the exogenous addition of these non-physiologically relevant metals replaced a bound ion. In addition, the co-crystallization of GspB_BR_ with α-2,3-sialyl (1-thioethyl)galactose disaccharide required crystallization conditions that included Ni^2+^, Cd^2+^, Co^2+^, and Mg^2+^, each at 7.5 mM. Anomalous dispersion experiments confirmed that Cd^2+^ was bound to the seven-coordinate site in these crystals.

**Synthesis of the α-2,3-sialyl (1-thioethyl)galactose disaccharide** The synthesis of α-2,3-sialyl (1-thioethyl)galactose, shown in **Scheme S1**, followed a modification of a synthesis of 2,3-STF reported by Danishefsky and co-workers .


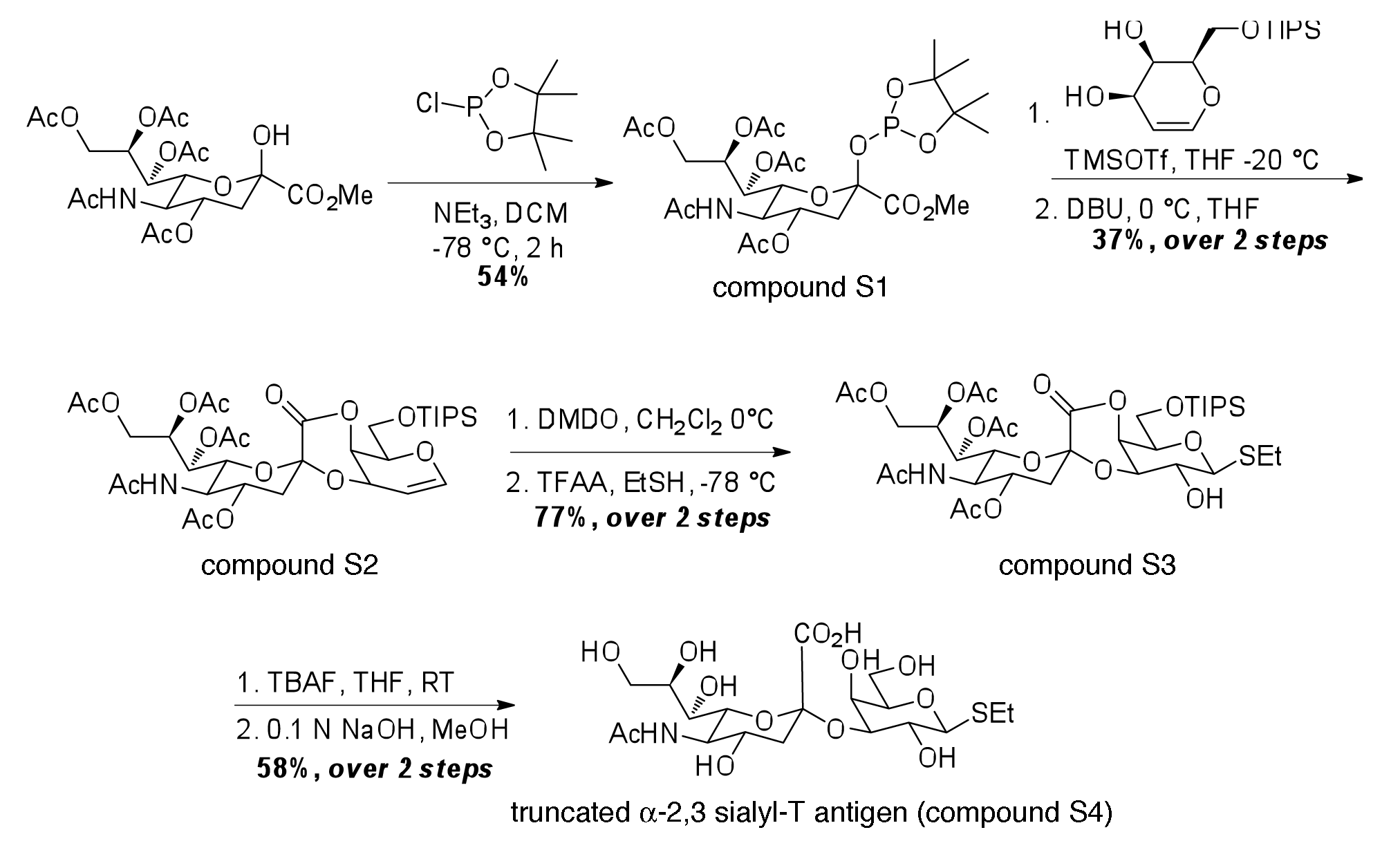


**Scheme S1. Synthesis of the** α**-2,3-sialyl (1-thioethyl)galactose disaccharide.**

*Synthesis of (1S,2R)-1-((2R,3R,4S,6R)-3-acetamido-4-acetoxy-6-(methoxycarbonyl)-6-((4,4,5,5-tetramethyl-1,3,2-dioxaphospholan-2-yl)oxy)tetrahydro-2H-pyran-2-yl)propane-1,2,3-triyl triacetate (compound* ***S1****).* To a solution of (1S,2R)-1-((2R,3R,4S,6S)-3-acetamido-4-acetoxy-6-hydroxy-6-(methoxycarbonyl)tetrahydro-2H-pyran-2-yl)propane-1,2,3-triyl triacetate (1.17 g, 2.38 mmol) in dichloromethane at -78 °C was added triethylamine (2.35 mL, 16.9 mmol) followed by a dropwise addition of 2-chloro-4,4,5,5-tetramethyl-1,3,2-dioxaphospholane (0.76 mL, 4.77 mmol). The solution was stirred at -78 °C for 1 h followed by dilution with ethyl acetate (80 mL) and saturated NaHCO_3_ (25 mL). The aqueous layer was extracted with ethyl acetate (2 X 40 mL) and the organic layers were combined. The organic layer was washed with brine (1 X 40 mL), dried over Na_2_SO_4_, filtered and concentrated. The resulting yellow oil was taken up in hexanes/DCM (7:1) and purified on Biotage silica gel chromatography (2% NEt_3_ in hexanes: EtOAc 25G SNAP Cartridge) to yield a pale yellow oil (824 mg, 54%, compound **S1**) that was used directly in the next reaction. Note: the compound was stored in DCM (ca. 30 mL) at -20 °C. [α]^23^_D_ -0.5° (*c* 7.88, CHCl_3_); ^1^H NMR (400MHz, CDCl_3_, δ(ppm)): 5.14 (dd; *J* = 5.2, 2.4Hz; 1H), 5.30 (d; *J* = 10Hz; 1H), 5.21-5.15 (m; 2H), 4.55 (dd; *J* = 8.4, 2.8Hz; 1H), 4.24-4.10 (m; 3H), 3.83 (s, 3H), 2.12 (s; 3H), 2.07 (s; 3H), 2.02 (s; 3H), 2.00 (s; 3H), 1.88 (s; *3*H), 1.50 (s; 3H), 1.39 (s; 3H), 1.26 (s; 3H), 1.23 (s; 3H). ^13^C NMR (100MHz, CDCl_3_, δ(ppm)): 170.9, 170.5, 170.4, 170.2, 170.1, 167.6, 97.4, 97.3, 85.4, 85.3, 72.0, 71.6, 68.7, 67.9, 62.1, 53.1, 49.3, 38.1, 25.2, 25.1, 24.9, 24.6, 23.1, 20.9, 20.8, 20.7. ^31^P NMR (162 MHz, CDCl_3_, δ(ppm)): 142.3.

*Synthesis of (1S,2R)-1-((4S,4a'R,5R,5'R,6R)-5-acetamido-4-acetoxy-3'-oxo-5'-(((triisopropylsilyl)oxy)methyl)-3,3',4,4a',5,5',6,8a'-octahydrospiro[pyran-2,2'-pyrano[3,4-b][1,4]dioxin]-6-yl)propane-1,2,3-triyl triacetate (compound* ***S2****), and (1S,2R)-1-((4S,4a'S,5R,5'R,6R,7'S,8'R,8a'R)-5-acetamido-4-acetoxy-7'-(ethylthio)-8'-hydroxy-3'-oxo-5'-(((triisopropylsilyl)oxy)methyl)decahydrospiro[pyran-2,2'-pyrano[3,4-b][1,4]dioxin]-6-yl)propane-1,2,3-triyl triacetate (compound* ***S3****):* Compounds S2 and S3 were prepared according to the literature precedent from compound **S1** .

*Synthesis of (2S,4S,5R,6R)-5-acetamido-2-(((2S,3R,4S,5S,6R)-2-(ethylthio)-3,5-dihydroxy-6-(hydroxymethyl)
tetrahydro-2H-pyran-4-yl)oxy)-4-hydroxy-6-((1R,2R)-1,2,3-trihydroxypropyl)tetrahydro-2H-pyran-2-carboxylic acid (compound* ***S4****; α-2,3-sialyl (1-ethylthio)galactose):* To a solution of lactone **S3** (113 mg, 0.13 mmol) in THF (1.3 mL) at ambient temperature was added TBAF solution (0.15 mL, 1M in THF) and stirred for 30 minutes. Once the reaction was complete by TLC analysis, the reaction was diluted with ethyl acetate (20 mL) and saturated NaHCO_3_ solution (10 mL). The mixture was transferred to a separatory funnel and the layers separated. The aqueous layer was washed with ethyl acetate (2 X 20mL) and the organic layers were combined. The organic layer was washed with brine (2 X 10 mL) and dried over Na_2_SO_4_, filtered and concentrated. The residue was dissolved in MeOH (1.3 mL) and 0.1 N NaOH solution (1.3 mL) was added. The solution was stirred for 24 h at ambient temperature. DOWEX 50WX4-100 strong cation exchange resin was added to lower the solution to pH 4. The mixture was filtered through an ISOLUTE SPE single fritted column to remove the resin and concentrated by lyophilization of water, resulting in a white crystalline solid confirmed to be the sialyl galactose disaccharide (compound **S4**) (41.8 mg, 58% over 2 steps). [α]^23^_D_-16.2 (*c* 3.16, CH_3_OH); ^1^H NMR (400MHz, CDCl_3_, δ(ppm)): 4.55 (d; *J* = 9.6Hz; 1H), 4.13 (dd; *J* = 9.6, 3.2Hz; 1H), 4.00 (d; *J* = 3.2Hz; 1H), 3.92-3.82 (m; 4H), 3.81-3.56 (m, 10H), 2.84-2.68 (m, 3H), 2.03 (s; 3H), 1.89 (t; *J* = 12.4Hz; 1H), 1.27 (t; *J* = 7.6Hz; 3H). ^13^C NMR (100MHz, CDCl_3_, δ(ppm)): 174.9, 172.6, 99.2, 85.0, 78.5, 76.9, 72.9, 71.2, 68.0, 67.8, 62.7, 60.8, 51.5, 48.8, 39.1, 23.9, 21.9, 14.3. HRMS calculated for C_19_H_33_NO_13_SNa (M+Na)^+^ *m/z* : 538.1570, measured 538.1570.

**SUPPORTING REFERENCES**

1. Schwarz JB, Kuduk SD, Chen XT, Sames D, Glunz PW, et al. (1999) A broadly applicable method for the efficient synthesis of alpha-O-linked glycopeptides and clustered sialic acid residues. Journal of the American Chemical Society 121: 2662-2673.
